# Pentanuclear iron catalysts for water oxidation: substituents provide two routes to control onset potentials†
†Electronic supplementary information (ESI) available: Synthetic details, characterization, crystal structures and experimental details. CCDC 1872481 and 1872482. For ESI and crystallographic data in CIF or other electronic format see DOI: 10.1039/c9sc00678h


**DOI:** 10.1039/c9sc00678h

**Published:** 2019-03-19

**Authors:** Vijayendran K. K. Praneeth, Mio Kondo, Masaya Okamura, Takuya Akai, Hitoshi Izu, Shigeyuki Masaoka

**Affiliations:** a Department of Life and Coordination-Complex Molecular Science , Institute for Molecular Science (IMS) , 5-1 Higashiyama, Myodaiji , Okazaki , Aichi 444-8787 , Japan . Email: masaoka@ims.ac.jp; b SOKENDAI [The Graduate University for Advanced Studies] , Shonan Village , Hayama , Kanagawa 240-0193 , Japan; c ACT-C , Japan Science and Technology Agency (JST) , 4-1-8 Honcho , Kawaguchi , Saitama 332-0012 , Japan; d Research Center of Integrative Molecular Systems (CIMoS) , Institute for Molecular Science (IMS) , 38 Nishigo-naka, Myodaiji , Okazaki , Aichi 444-8585 , Japan

## Abstract

The development of robust and efficient molecular catalysts based on earth-abundant transition metals for water oxidation reactions is a challenging research target. Our group recently demonstrated the high activity and stability of a pentairon-based water oxidation electrocatalyst (M. Okamura, M. Kondo, R. Kuga, Y. Kurashige, T. Yanai, S. Hayami, V. K. K. Praneeth, M. Yoshida, K. Yoneda, S. Kawata and S. Masaoka, *Nature*, 2016, **530**, 465–468). However, the development of strategies to decrease onset potentials for catalysis remains challenging. In this article, we report the construction of a series of pentanuclear iron complexes by introducing electron-donating (methyl) and electron-withdrawing (bromo) substituents on the ligand. Two newly synthesized complexes exhibited five reversible redox processes, similar to what is seen with the parent complex. These complexes can also serve as homogeneous catalysts for water oxidation reactions, and the faradaic efficiencies of the reactions were high. Additionally, the onset potentials of the newly developed complexes were lower than that of the parent complex. Mechanistic insights revealed that there are two methods for decreasing onset potentials: control of the redox potentials of the pentairon complex and control of the reaction mechanism.

## Abstract 2

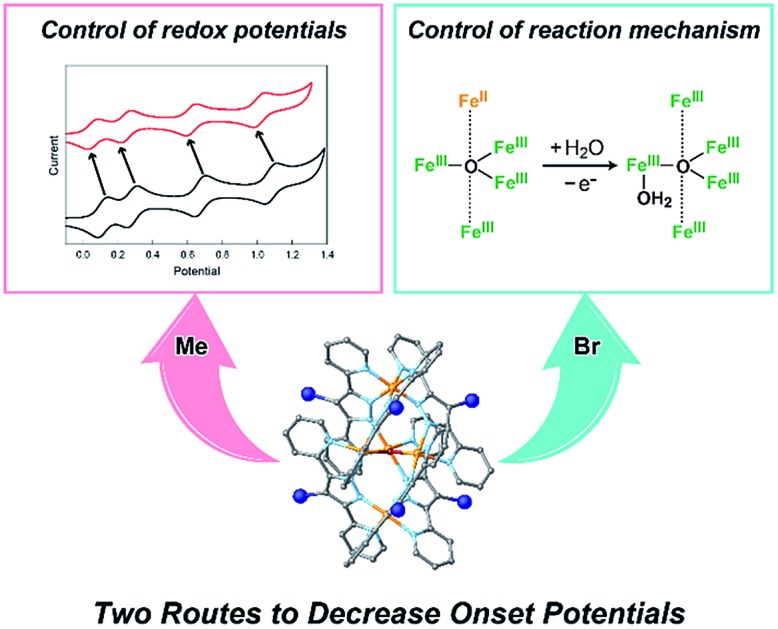
Two distinct routes to decrease the onset potential for water oxidation were provided by either control of redox potentials of the complex or change of the reaction mechanism in the pentairon catalysts. The results offer a novel strategy to design efficient molecule-based catalysts for water oxidation.

## Introduction

Water oxidation (2H_2_O → O_2_ + 4H^+^ + 4e^–^) is considered the main bottleneck in the production of chemical fuels from sunlight and/or electricity;^1^–^7^ this is because the reaction requires the transfer of four electrons and the generation of an O–O bond, and is both thermodynamically and kinetically demanding. Therefore, the development of a highly active artificial catalyst for the oxidation of water is of great importance. In this context, since the discovery of the first molecular water oxidation catalyst, “Blue dimer”,^8^ a significant number of molecular water oxidation catalysts have been reported.^9^–^24^ More recently, metal complexes containing earth-abundant transition metal ions such as Mn,^25^–^28^ Fe,^29^–^36^ Co,^37^–^46^ and Cu^47^–^55^ ions have also been intensely studied. Nevertheless, the development of efficient metal-complex-based catalysts that consist of earth-abundant transition metals is still very challenging.^56^,^57^

In nature, water oxidation is catalysed by the oxygen evolving complex (OEC) in photosystem II.^58^–^60^ The OEC is a highly active and robust catalyst for water oxidation that can drive the reaction under mild conditions.^61^ The active site of the OEC contains a multinuclear metal complex, a Mn_4_CaO_5_ cluster, which has several water coordination sites. Due to the multinuclear structure of the Mn_4_CaO_5_ cluster, the OEC can smoothly accumulate the oxidative equivalents required for the reaction *via* the formation of five distinct redox intermediates, the S_*n*_ states, where the subscript indicates the number of stored oxidative equivalents (*n* = 0–4). After the formation of the **S_4_** state, H_2_O reacts with the **S_4_** state to generate O_2_ and protons.^62^

**Scheme 1:**
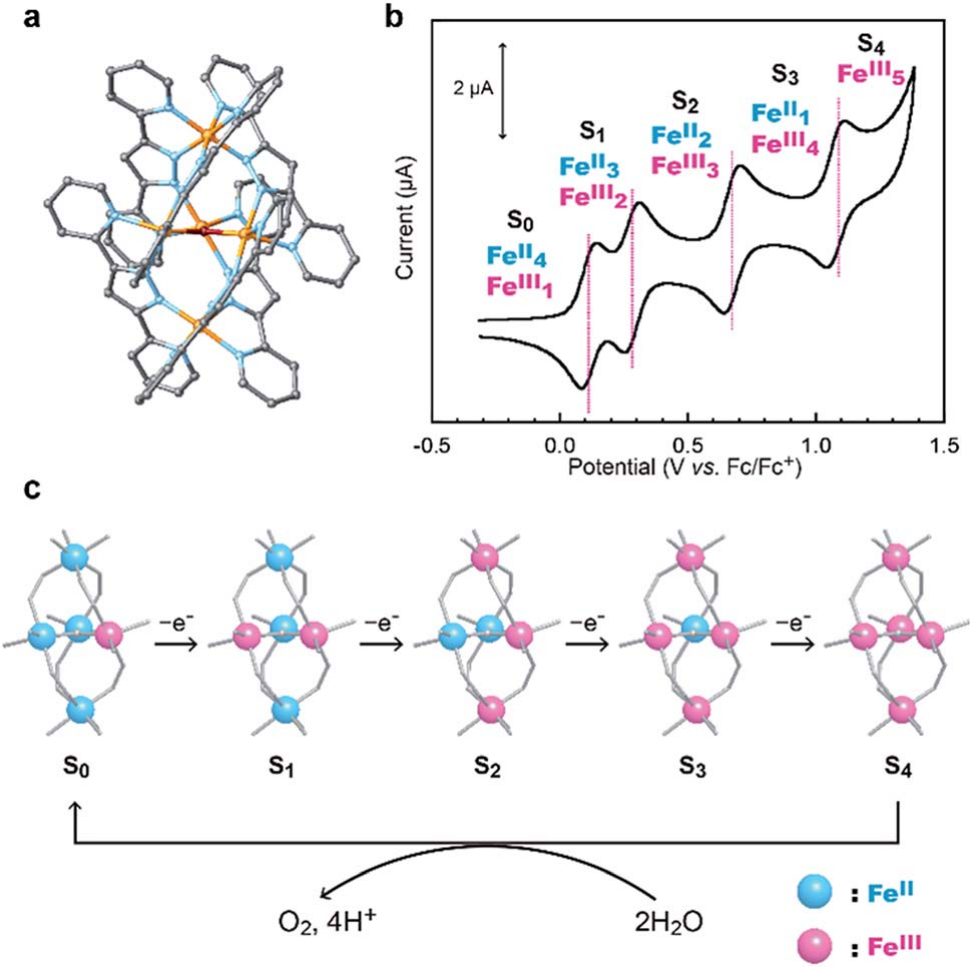
(a) Chemical structure of **[Fe_5_-H]^3+^**, (b) a cyclic voltammogram of **[Fe_5_-H]^3+^** (0.2 mM in MeCN solution containing 0.1 M TBAP under Ar at a scan rate of 10 mV s^–1^) and (c) sequential oxidation of iron ions in **[Fe_5_-H]^3+^** and the reaction of the **S_4_** state to generate dioxygen.

Recently, we demonstrated that a pentanuclear iron complex [FeII4Fe^III^(μ_3_-O)(bpp)_6_]^3+^, **[Fe_5_-H]^3+^** (Scheme 1a, Hbpp = bis(pyridyl)pyrazole), can serve as a highly active catalyst for electrocatalytic water oxidation.^33^**[Fe_5_-H]^3+^** can also accumulate four oxidative equivalents *via* the successive oxidation of each of the iron centres in the complex (Scheme 1b). In the catalysis mediated by **[Fe_5_-H]^3+^**, the four-electron-oxidized species, [FeIII5(μ_3_-O)(bpp)_6_]^7+^ (**[Fe_5_-H]^7+^**, **S_4_** state), reacts with H_2_O to generate O_2_ (Scheme 1c). The reaction rate and durability of **[Fe_5_-H]^3+^** are the highest among those of iron-based water oxidation catalysts (Fe-WOCs) reported thus far. However, a relatively large onset potential is required for the catalysis because the **S_4_** state is only generated at high potentials. Therefore, the development of a novel strategy for designing catalysts that can drive the reaction at low onset potentials is essential.

**Scheme 2:**
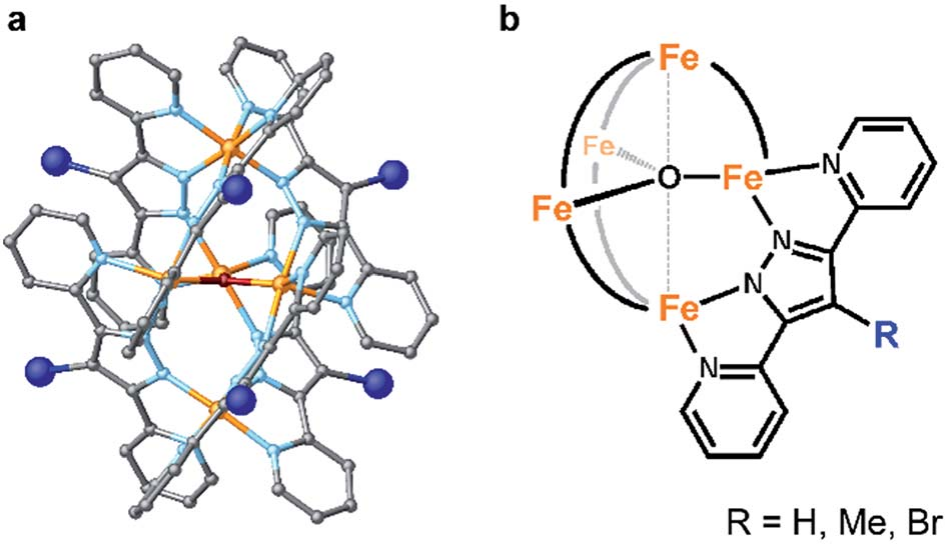
Schematic illustrations of the series of pentanuclear iron complexes bearing 4-substituted-3,5-bis(pyridyl)pyrazole (R-Hbpp (R = H, Me, and Br)) ligands investigated in this study.

Here, we report two approaches for decreasing the onset potential of pentairon water oxidation systems. Two approaches involving the installation of substituents onto the Hbpp ligand have been demonstrated. Two kinds of ligands, one with electron-donating and the other with electron-withdrawing groups at the 4-position of the Hbpp ligand (Me-Hbpp and Br-Hbpp in Scheme 2), have been employed, and the new pentairon complexes were constructed utilizing these ligands. The newly synthesized complexes catalysed the oxidation of water with high faradaic efficiencies, and the onset potentials of these complexes were lower than that of the parent complex. The mechanistic studies also revealed that two distinct routes exist to decrease the onset potentials for water oxidation in pentanuclear iron systems.

## Results

### Syntheses and characterization of ligands and pentairon complexes

Me-Hbpp was prepared using the three-step synthetic route shown in Scheme S1 in the ESI.† Initially, 2-acetylpyridine and pyridine-2-carboxylic acid methyl ester were reacted in the presence of sodium ethoxide to yield 1,3-di(2-pyridyl)-1,3-propanedione. The methylation of the product and further treatment with hydrazine monohydrate afforded Me-Hbpp. The total synthetic yield of Me-Hbpp was 26% (see the Experimental section for details). Br-Hbpp was synthesized in a moderate yield (65%) by the bromination of Hbpp.^63^ Both ligands were characterized by ^1^H and ^13^C NMR spectroscopy and elemental analyses. The syntheses of two pentairon complexes with the obtained ligands were performed by reacting the corresponding ligand (6 eq.) with FeSO_4_·7H_2_O (5 eq.) in the presence of a base (NaOH, 6 eq.) in methanol at 80 °C (Scheme S2†). The reaction mixture was further treated with a saturated solution of aqueous NaBF_4_ or NaPF_6_, and the obtained precipitate was collected by filtration. The precipitate was recrystallized from MeCN/Et_2_O to afford crystalline products. The electrospray ionization mass spectrometry (ESI-MS) and elemental analysis data of the obtained crystalline samples confirmed the formation of the desired pentairon complexes, [FeII4Fe^III^(μ_3_-O)(Me-bpp)_6_]^3+^ (**[Fe_5_-Me]^3+^**) and [FeII4Fe^III^(μ_3_-O)(Br-bpp)_6_]^3+^ (**[Fe_5_-Br]^3+^**). The synthetic yields of the complexes were 62 and 43% for **[Fe_5_-Me]^3+^** and **[Fe_5_-Br]^3+^**, respectively. The parent complex, **[Fe_5_-H]^3+^**, was synthesized using a reported procedure^33^ and characterized by ESI-MS and elemental analysis.

### Crystal structures of the pentairon complexes

**Fig. 1:**
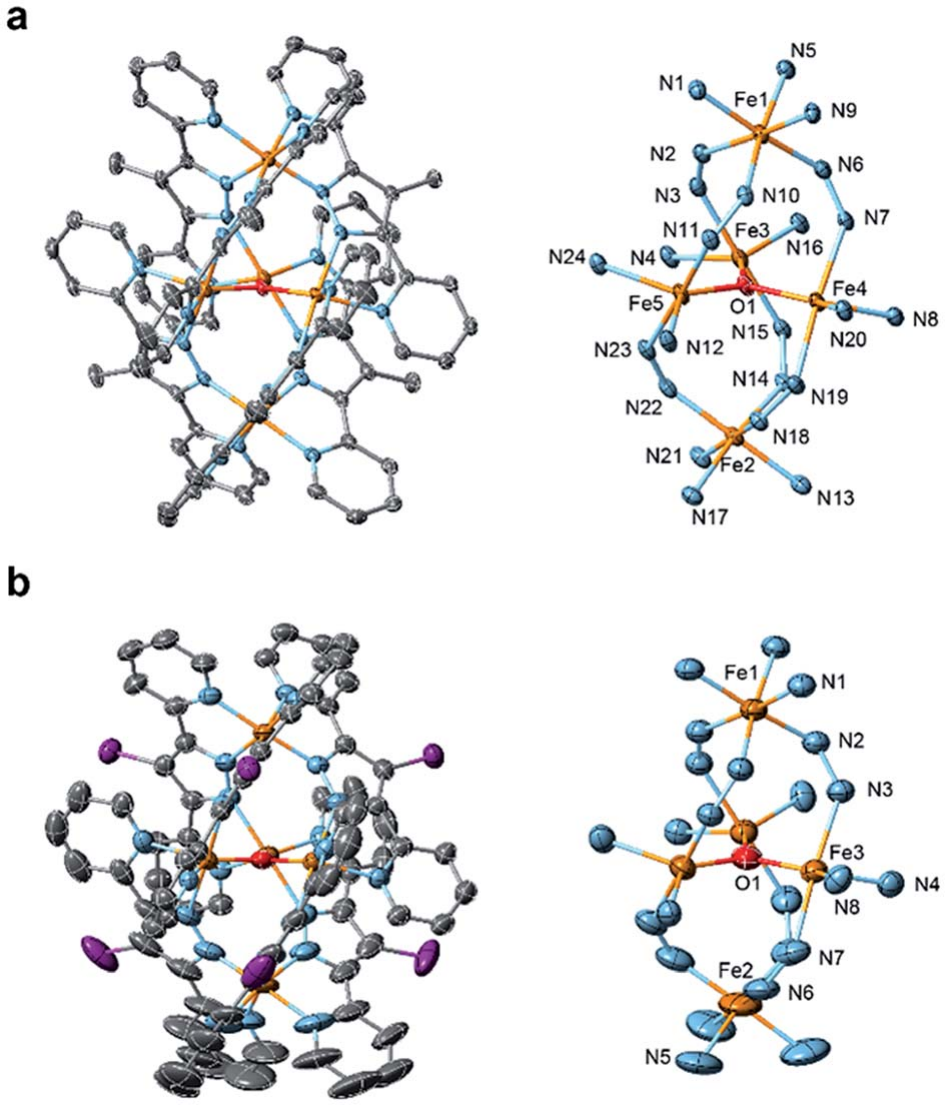
ORTEP drawings of the cationic moieties (left) and core structures (right) of (a) **[Fe_5_-Me]**(PF_6_)_3_ and (b) **[Fe_5_-Br]**(BF_4_)_3_. The atoms are represented by the following colours: Fe, orange; O, red; N, blue; C, grey; and Br, purple. Hydrogen atoms and crystal solvent molecules are omitted for clarity. Thermal ellipsoids are shown at 30% probability.

Single crystals of **[Fe_5_-Me]^3+^** and **[Fe_5_-Br]^3+^** suitable for single-crystal X-ray diffraction (SCXRD) were obtained by vapor diffusion of diethyl ether (Et_2_O) into saturated solutions of the respective complexes in acetonitrile (MeCN). Note that a few drops of MeOH were added to the MeCN solution of **[Fe_5_-Me]^3+^** to prevent the oxidation of the complex. As we previously reported,^33^ single crystals of **[Fe_5_-H]^3+^** were obtained by slow evaporation of acetonitrile from a 1 : 1 MeCN–H_2_O (v/v) solution of the complex. **[Fe_5_-Me]^3+^** was obtained as a PF_6_ salt, and **[Fe_5_-Br]^3+^** and **[Fe_5_-H]^3+^** were obtained as BF_4_ salts. The ORTEP diagrams of the cationic moieties of the three complexes are shown in Fig. 1 and S1,† and the crystallographic data for the newly synthesized complexes are summarized in Table S1.† All complexes displayed the same structural motif consisting of a central [Fe_3_(μ_3_-O)] core connected by two apical Fe ions through six R-bpp^–^ units. **[Fe_5_-Me]^3+^** crystallized in the *P*1[combining macron] space group, and the asymmetric unit contains one cationic pentairon complex and three PF_6_ anions. The asymmetric unit of the *R*3[combining macron] crystal of **[Fe_5_-Br]^3+^** is composed of one-third of the cationic pentairon complex and one BF_4_ anion. The crystal structure of **[Fe_5_-H]^3+^** belongs to the *I*4[combining macron] space group, and the asymmetric unit contains half of the cationic pentairon complex and one and half BF_4_ anions. The bond distances between the iron atoms and the N atoms on R-bpp^–^ ligands are not significantly changed by either bromo or methyl substitution (Table S2†). These results clearly demonstrate that the substituents do not affect the pentanuclear core structure of the complexes (Fig. S2†).

### UV-Vis absorption spectra

The UV-Vis absorption spectra of **[Fe_5_-Me]^3+^**, **[Fe_5_-Br]^3+^** and **[Fe_5_-H]^3+^** in MeCN are shown in Fig. S3.† The complexes exhibited intense bands at approximately 400–410 nm and shoulder peaks at 480–490 nm. These bands are attributed to the charge transfer from the low-spin Fe^II^ centre to the ligand (MLCT).^33^,^64^ In our previous study, Mössbauer spectroscopic measurements of **[Fe_5_-H]^3+^** showed that the two iron centres at the apical positions are low-spin Fe^II^ ions and that the [Fe_3_(μ_3_-O)] core is composed of two high-spin Fe^II^ ions and one high-spin Fe^III^ ion.^33^ The UV-Vis absorption spectra strongly indicate that the oxidation and spin states of the iron centres of **[Fe_5_-Me]^3+^** and **[Fe_5_-Br]^3+^** are identical to those of **[Fe_5_-H]^3+^**. Note that the MLCT bands of **[Fe_5_-Me]^3+^** and **[Fe_5_-Br]^3+^** are slightly red shifted and blue shifted, respectively, compared to those of **[Fe_5_-H]^3+^**, reflecting the electron-donating or electron-withdrawing nature of the substituents.

### Electrochemical properties

**Fig. 2:**
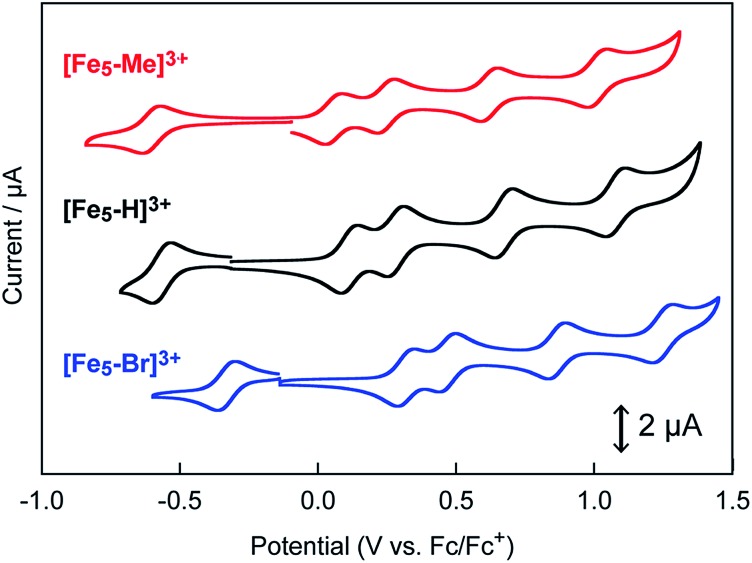
Cyclic voltammograms of 0.2 mM solutions of **[Fe_5_-Me]^3+^** (red line), **[Fe_5_-Br]^3+^** (blue line) and **[Fe_5_-H]^3+^** (black line). Measurements were performed in MeCN solutions containing 0.1 M TBAP under Ar at a scan rate of 10 mV s^–1^. Working electrode, glassy carbon; counter electrode, Pt wire; reference electrode, Ag/Ag^+^. CV scans were initiated from the respective open-circuit potentials.

The influence of the electron-donating and electron-withdrawing groups on the redox properties of the pentairon complexes was investigated by cyclic voltammetry. The cyclic voltammograms (CVs) of **[Fe_5_-Me]^3+^**, **[Fe_5_-Br]^3+^** and **[Fe_5_-H]^3+^** (0.2 mM) in dry MeCN containing 0.1 M Bu_4_NClO_4_ (TBAP) under an Ar atmosphere are shown in Fig. 2, and the electrochemical data are summarized in Table 1. All the complexes displayed one reversible and four successive reversible one-electron oxidation waves assigned to the FeII4Fe^III^/FeII5, FeII3FeIII2/FeII4Fe^III^, FeII2FeIII3/FeII3FeIII2, Fe^II^FeIII4/FeII2FeIII3, and FeIII5/Fe^II^FeIII4 redox couples, indicating that the electron transfer ability arising from the pentairon structure is preserved even after the introduction of substituents on the ligands. Importantly, all the redox waves of **[Fe_5_-Me]^3+^** were shifted to a more negative potential relative to those of **[Fe_5_-H]^3+^**, whereas the redox waves of **[Fe_5_-Br]^3+^** were positively shifted (Fig. 2 and Table 1). These trends are consistent with the electron-donating and electron-withdrawing properties of the methyl and bromo substituents, respectively. This result clearly demonstrates that the redox potentials of the pentairon complexes can be tuned by the introduction of substituents on the ligands. The open-circuit potentials of the complexes, located at –0.10 (**[Fe_5_-Me]^3+^**), –0.18 (**[Fe_5_-Br]^3+^**), and –0.26 V (**[Fe_5_-H]^3+^**), indicate an initial state of FeII4Fe^III^ in solution. These initial oxidation states of the complexes in the solution state determined by the electrochemical measurements are fully consistent with those estimated from the UV-Vis absorption spectra (*vide supra*).

**Table 1:** Redox potentials (*E*_1/2_, V *vs.* Fc/Fc^+^) and the onset potentials for water oxidation (*E*_onset_, V *vs.* Fc/Fc^+^) of a series of pentairon complexes in acetonitrile solutions with TBAP (0.1 M). *E*_1/2_(–1), *E*_1/2_(1), *E*_1/2_(2), *E*_1/2_(3), and *E*_1/2_(4) correspond to the *E*_1/2_ values of the FeII4Fe^III^/FeII5, FeII3FeIII2/FeII4Fe^III^, FeII2FeIII3/FeII3FeIII2, Fe^II^FeIII4/FeII2FeIII3, and FeIII5/Fe^II^FeIII4 redox couples, respectively

Complex	*E*_1/2_					*E*_onset_ ^*a*^
	*E*_1/2_(–1)	*E*_1/2_(1)	*E*_1/2_(2)	*E*_1/2_(3)	*E*_1/2_(4)	
**[Fe_5_-Me]^3+^**	–0.60	0.05	0.24	0.62	1.01	1.09
**[Fe_5_-Br]^3+^**	–0.32	0.32	0.48	0.87	1.27	1.15
**[Fe_5_-H]^3+^**	–0.55	0.13	0.30	0.68	1.08	1.18

^*a*^Conditions: [cat] = 0.2 mM, [H_2_O] = 5 M, working electrode: glassy carbon, scan rate: 10 mV s^–1^.

### Catalytic activity for water oxidation

**Fig. 3:**
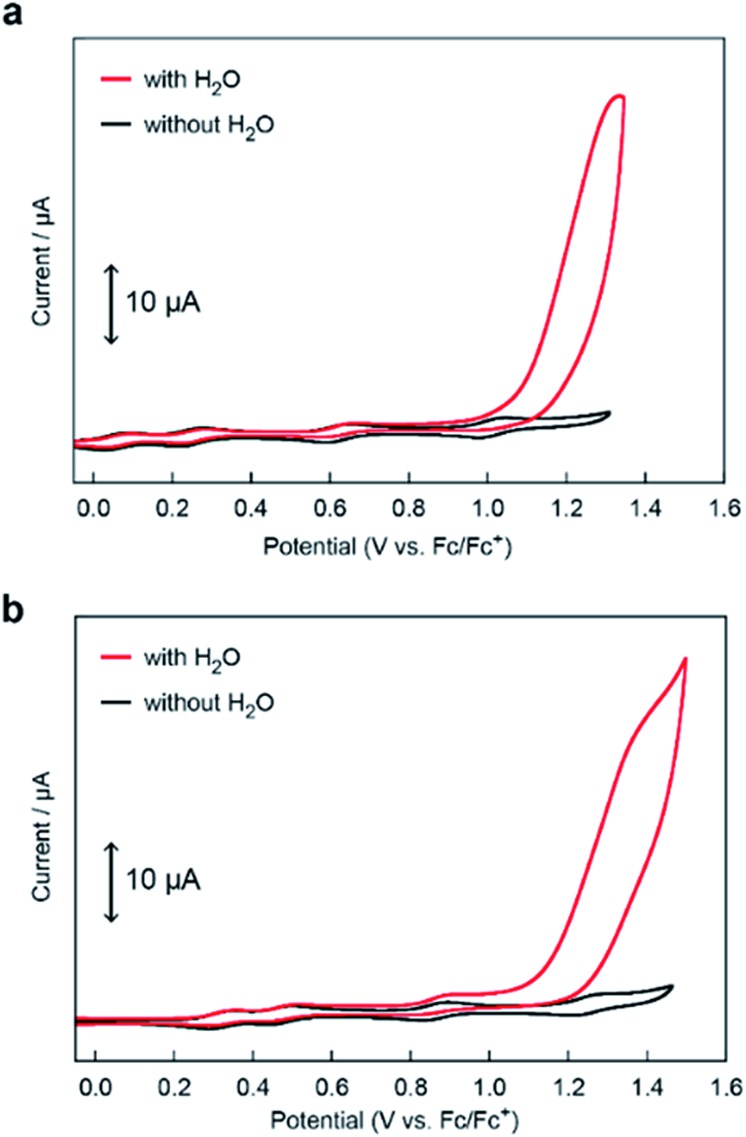
CVs of 0.2 mM solutions of (a) **[Fe_5_-Me]^3+^** and (b) **[Fe_5_-Br]^3+^** in MeCN containing 0.1 M TBAP in the absence of H_2_O (black lines) and the presence of 5 M H_2_O (pH = 5, red lines). The CVs were measured using a GC electrode under an Ar atmosphere at a scan rate of 10 mV s^–1^.

**Fig. 4:**
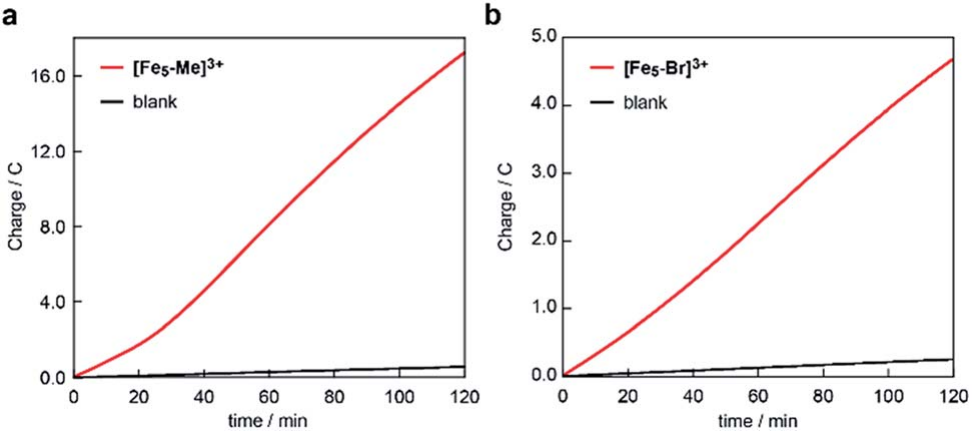
Controlled potential electrolysis data of (a) **[Fe_5_-Me]^3+^** and (b) **[Fe_5_-Br]^3+^** in a MeCN/H_2_O mixed solution system containing 0.1 M TBAP using an ITO electrode under an argon atmosphere. Conditions: [cat] = 0.2 mM, [H_2_O] = 5 M and applied potential: 1.42 V *vs.* Fc/Fc^+^, pH = 5.

The electrocatalytic activities of **[Fe_5_-Me]^3+^** and **[Fe_5_-Br]^3+^** for water oxidation were examined by electrochemical measurements of the 0.2 mM solution of the complexes in the presence of 5 M H_2_O. Under these conditions, both complexes exhibited a large irreversible current in the >1.0 V region (Fig. 3). As previously reported for **[Fe_5_-H]^3+^**,^33^ such an increase in the current is indicative of promotion of the electrocatalytic water oxidation reaction. To further verify whether the electrocatalytic current actually corresponds to the activity in the catalytic oxidation of H_2_O, controlled potential electrolysis (CPE) was carried out at an indium tin oxide (ITO) electrode using a customized two-compartment cell system.^33^ After 2 h of electrolysis of **[Fe_5_-Me]^3+^** at 1.42 V (*vs.* Fc/Fc^+^), 17.2 C of charge was passed, and gas chromatography (GC) detected 39.5 μmol of O_2_ as the product (Fig. 4a). In the case of **[Fe_5_-Br]^3+^**, the electrolysis under identical conditions afforded a charge of 4.7 C and generated 9.3 μmol of O_2_ (Fig. 4b). The faradaic efficiencies of the reaction based on the 4e^–^ process were 92 and 86% for **[Fe_5_-Me]^3+^** and **[Fe_5_-Br]^3+^**, respectively. Based on the results of CPE experiments, turnover frequencies (TOFs) and turnover numbers (TONs) for water oxidation were roughly estimated. For **[Fe_5_-Me]^3+^**, TOF and TON were 3 × 10^2^ s^–1^ and 2 × 10^6^, respectively, and TOF and TON values of **[Fe_5_-Br]^3+^** were estimated to be 20 s^–1^ and 1 × 10^5^, respectively (for the details of calculation see the ESI (P.S24)†). Although these values were lower than those of **[Fe_5_-H]^3+^** (1 × 10^3^ s^–1^ (TOF) and 7.5 × 10^6^ (TON)) estimated by using the same method, they were substantially higher compared to those of the reported iron-complex-based catalysts for water oxidation.^29^–^32^,^35^ In both cases, the electrolyzed solutions were treated with oxo[5,10,15,20-tetra(4-pyridyl)porphyrinato]titanium(iv) as a chemical probe^65^ and the 2e^–^ oxidized product of H_2_O (H_2_O_2_) was not detected (for details of the experimental procedure, see the ESI (P.S22–S23)†). After the CPE experiment, the ITO working electrodes used in the electrolysis were gently rinsed with small amounts of water and MeCN, and then, a second round of electrolysis was performed using the solution without the catalyst. Significantly small currents were observed in the second electrolysis compared to the first electrolysis in both cases (Fig. S4 and S5†), which indicates that the species homogeneously dissolved in the solution are catalytically active. CV measurements of the solution after the CPE experiments also clarified the presence of pentanuclear complexes in the solution phase (Fig. S6†). Additionally, the UV-Vis absorption spectra of the ITO electrodes before and after the CPE experiments remained almost identical (Fig. S7 and S8†), suggesting no formation of heterogeneous deposits during the electrolysis. We also analysed the electrolyte solutions after the electrolysis by dynamic light scattering (DLS) measurements and no formation of heterogeneous nanoparticles was detected (Fig. S9†). These experimental results show that **[Fe_5_-Me]^3+^** and **[Fe_5_-Br]^3+^** can serve as homogeneous electrocatalysts for water oxidation.

### Onset potentials for water oxidation

Although both of the novel pentanuclear complexes, **[Fe_5_-Me]^3+^** and **[Fe_5_-Br]^3+^**, exhibited catalytic activity for water oxidation, the electrochemical responses of these complexes in the presence of H_2_O were different. In the case of **[Fe_5_-Me]^3+^**, a large irreversible current attributed to the catalytic water oxidation was observed at a potential close to that of the fourth redox couple (FeIII5/Fe^II^FeIII4, *E*_1/2_(4) in Table 1) in the presence of 5 M H_2_O (Fig. 3a). The onset potential (*E*_onset_, Table 1) for the reaction was estimated from the cross-point of two lines that were obtained by extrapolating the slopes of the catalytic current and non-faradaic current. The *E*_onset_ of **[Fe_5_-Me]^3+^** was determined to be 1.09 V, which corresponds to the overpotential (*η*) of 0.65 V at pH = 5.0, and was slightly larger than *E*_1/2_ of the fourth redox couple (1.01 V). In contrast, the catalytic current for water oxidation for **[Fe_5_-Br]^3+^** was observed at a more negative potential than that of the fourth redox couple (1.27 V, Fig. 3b); the *E*_onset_ of **[Fe_5_-Br]^3+^** was determined to be 1.15 V (*η* = 0.71 V at pH = 5.0). These results clearly demonstrate that **[Fe_5_-Me]^3+^** and **[Fe_5_-Br]^3+^** catalyse the water oxidation reaction by distinct reaction mechanisms. Notably, the *E*_onset_ of these complexes is lower than that of **[Fe_5_-H]^3+^** (1.18 V, *η* = 0.74 V at pH = 5.0) under identical experimental conditions.

## Discussion

### Reaction mechanism of **[Fe_5_-Me]^3+^**

**Fig. 5:**
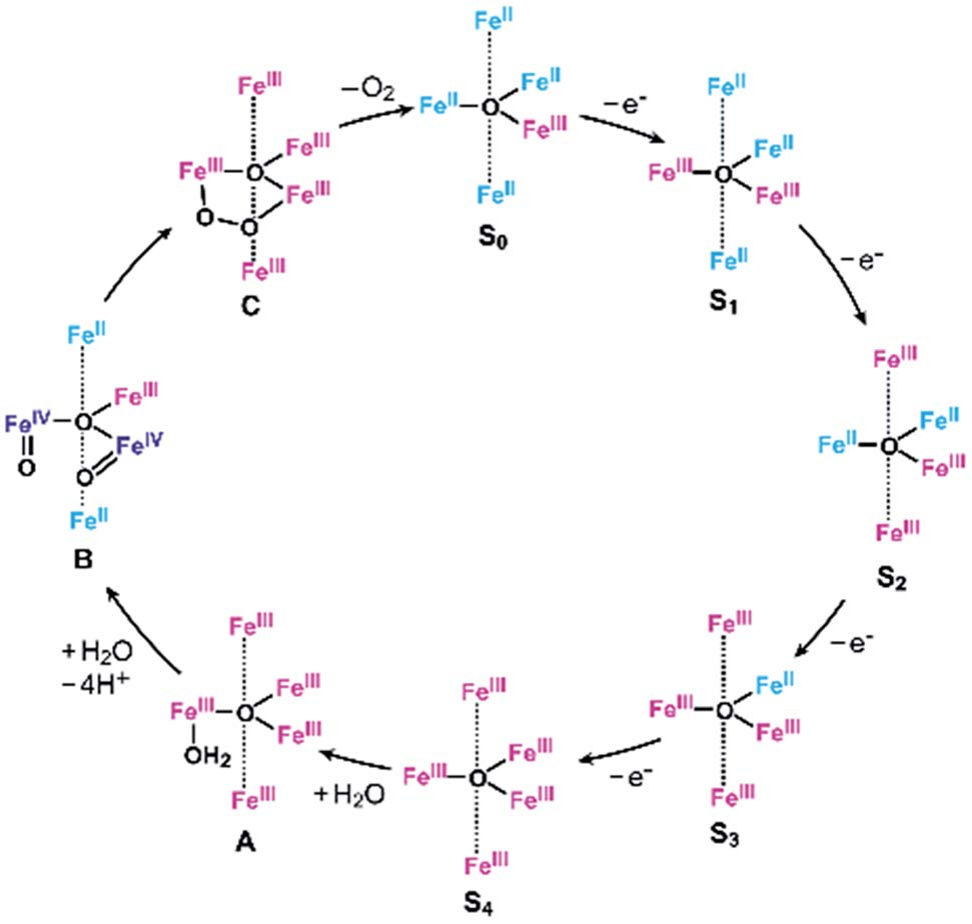
Proposed reaction mechanism for the water oxidation catalyzed by **[Fe_5_-Me]^3+^** and **[Fe_5_-H]^3+^**.^33^ Fe^II^, blue; Fe^III^, red; and Fe^IV^, purple.

As described above, the *E*_onset_ of **[Fe_5_-Me]^3+^** is located at a slightly more positive potential than the *E*_1/2_ of the fourth redox couple (Table 1). In other words, the formation of the four-electron oxidized species (FeIII5, the **S_4_** state) triggers the reaction with a water molecule and the subsequent oxidation of water in this case. A similar trend was also observed in the previously reported electrocatalysis by **[Fe_5_-H]^3+^**; the onset of the catalytic wave is coupled with the formation of the four-electron oxidized species. Therefore, it is suggested that **[Fe_5_-Me]^3+^** probably promotes electrocatalytic water oxidation through a catalytic cycle similar to that of **[Fe_5_-H]^3+^**, which we previously proposed based on experimental and computational studies^33^ (Fig. 5, see also the ESI (P.S32)†). In the catalytic cycle, the successive four-step, one-electron oxidation of the resting FeII4Fe^III^ (the **S_0_** state) initially generates the four-electron oxidized species FeIII5 (the **S_4_** state) *via* the **S_1_**, **S_2_** and **S_3_** states. In the **S_4_** state, all the iron atoms in the [Fe_3_(μ_3_-O)] core are in the Fe^III^ state. Subsequently, the addition of H_2_O to this fully oxidized [Fe_3_(μ_3_-O)] core of the **S_4_** state generates the water-bound FeIII5(OH_2_) species (intermediate **A**). Intermediate **A** then reacts with an additional H_2_O molecule to generate O_2_ and regenerate the initial **S_0_** state. Therefore, the shift of the *E*_onset_ of **[Fe_5_-Me]^3+^** (approximately 90 mV lower than that of **[Fe_5_-H]^3+^**) is attributed to the electron-donating nature of the methyl groups, which reduces the potential to generate the **S_4_** state.

### Reactivity of the **S_3_** state of **[Fe_5_-Br]^3+^**

Unlike **[Fe_5_-Me]^3+^** and **[Fe_5_-H]^3+^**, the catalytic current for **[Fe_5_-Br]^3+^** was not coupled with the fourth redox wave. Instead, the catalytic current (*E*_onset_ = 1.15 V) arises in the potential region between the third (0.87 V) and fourth (1.27 V) redox waves (*vide supra*). This result indicates that the Fe^II^FeIII4 state (the **S_3_** state) generated by the third redox process should undergo a chemical reaction (EC process), and the formed species should be further oxidized at 1.15 V. Alternatively, a concerted chemical and electrochemical process should occur at 1.15 V. In any case, the **S_3_** state is considered the key intermediate for water oxidation in the case of **[Fe_5_-Br]^3+^**.

**Fig. 6:**
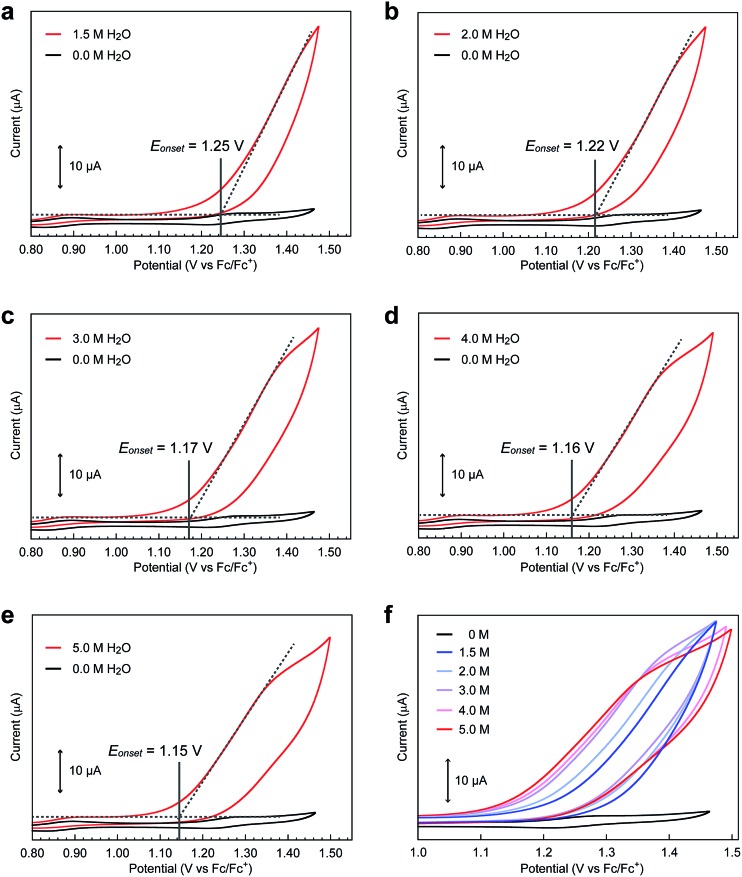
Cyclic voltammograms of **[Fe_5_-Br]^3+^** (0.2 mM) in the presence of (a) 1.5, (b) 2.0, (c) 3.0, (d) 4.0, and (e) 5.0 M H_2_O at pH = 5.0 (red lines) and in the absence of H_2_O (black lines). (f) Overlaid CVs of **[Fe_5_-Br]^3+^** at various concentrations of H_2_O. CVs were measured in acetonitrile solutions with TBAP (0.1 M) on a GC electrode at a scan rate of 10 mV s^–1^. The onset potentials were estimated from the cross-points of two lines, which are obtained by extrapolating the slopes of the catalytic current and non-faradaic current.

Intrigued by this finding, we set out to investigate the reactivity of the **S_3_** state of **[Fe_5_-Br]^3+^**. Initially, electrochemical measurements were performed at various concentrations of **[Fe_5_-Br]^3+^** to confirm if the **S_3_** state undergoes unimolecular or bimolecular reactions. As shown in Fig. S12,† the intensity of the catalytic peak current was linearly dependent on the concentration of **[Fe_5_-Br]^3+^**, suggesting that the rate is first order to the catalyst concentration, and therefore a bimolecular path requiring the association of catalysts is ruled out. Second, we hypothesized that the reaction of the **S_3_** state with H_2_O to form a H_2_O bound species and subsequent oxidation of the formed species is a possible pathway. To validate this hypothesis, CVs were collected at various scan rates in the presence of 5 M H_2_O by reversing the scan of potentials at 1.04 V. As shown in Fig. S13a,† the redox potentials (*E*_1/2_ values) and the wave shapes of the first three redox couples remained unchanged. Additionally, the reversibility of the third redox couple (Fe^II^FeIII4/FeII2FeIII3) was investigated by plotting the intensity of the anodic and cathodic peak currents against the square root of the scan rates (Fig. S13b†). The linearity of the obtained plot confirms that the third redox process is fully reversible and that no EC process occurs in this potential range. Therefore, the reaction of the **S_3_** state with H_2_O does not proceed in this potential region, and this pathway can be excluded. Third, the possibility of the **S_3_** state undergoing a proton-coupled electron transfer (PCET) reaction was considered because catalytic water oxidation reactions often involve such a process.^15^ However, this process hardly occurs because no dissociative proton exists in the **S_3_** state. Moreover, the CVs of **[Fe_5_-Br]^3+^** recorded under various pH conditions showed no change in the onset potential for water oxidation (Fig. S14†). Therefore, the **S_3_** state undergoing a PCET process is also unlikely. Finally, an electron transfer reaction coupled with water binding to the **S_3_** state was investigated. CVs of **[Fe_5_-Br]^3+^** at various concentrations of H_2_O were acquired. As shown in Fig. 6 and S15,† the onset potentials of the electrocatalytic current gradually shifted to lower potentials as the content of H_2_O increased. Note that the onset potential of water oxidation was not affected by the concentrations of H_2_O in the case of **[Fe_5_-H]^3+^** (Fig. S16†). This result clearly demonstrates that the electron transfer reaction coupled with the binding of H_2_O to the **S_3_** state is the key step in the **[Fe_5_-Br]^3+^**-catalysed reaction.

### Electronic structures of **S_3_** states

**Fig. 7:**
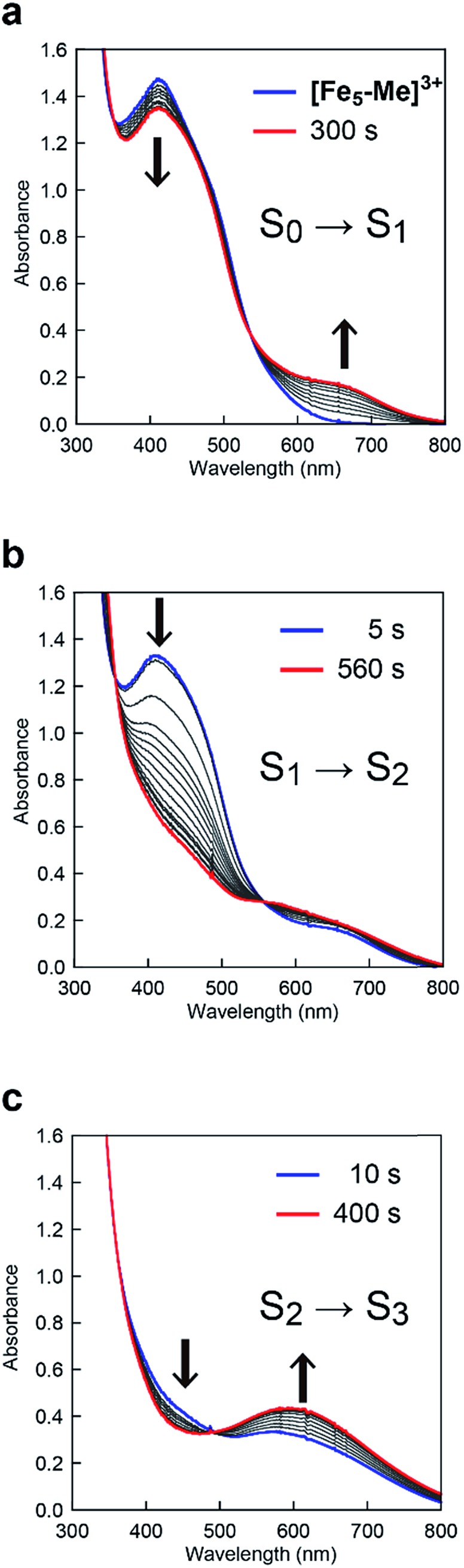
UV-Vis absorption spectra of **[Fe_5_-Me]^3+^** (0.05 mM) at various applied potentials ((a) 0.17, (b) 0.50 and (c) 0.86 V (*vs.* Fc/Fc^+^)) in 0.1 M TBAP/MeCN. Solutions were purged with Ar for 15 min prior to measurements. Weak Ar flow was maintained throughout the measurements.

**Scheme 3:**
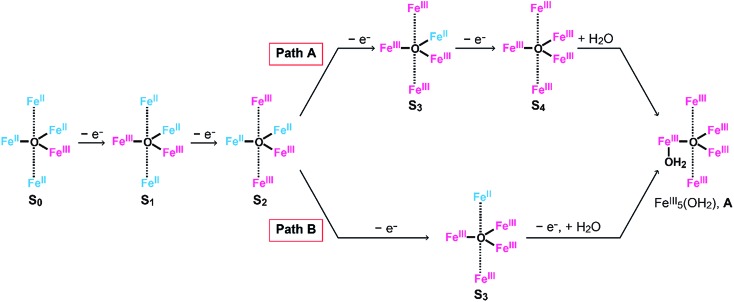
Oxidation processes of (Path A) **[Fe_5_-H]*^n^*^+^** and **[Fe_5_-Me]*^n^*^+^** and (Path B) **[Fe_5_-Br]*^n^*^+^**.

To clarify the origin of the unique reactivity of the **S_3_** state of **[Fe_5_-Br]^3+^**, the electronic structures of the **S_3_** states of a series of pentanuclear iron complexes were investigated. As we previously reported,^33^ the three-step oxidation of **[Fe_5_-H]^3+^** affords the **S_3_** state as evidenced by UV-Vis absorption spectroscopy (Fig. S17 and S18†). In the first step, a slight decrease in the MLCT band at 406 nm and the growth of a new broad peak at approximately 640 nm were observed. This newly observed peak is attributed to the formation of the [Fe^II^FeIII2(μ_3_-O)] central core.^33^ In other words, the first step corresponds to the oxidation of the central core, which yields [{Fe^II^(μ-bpp)_3_}_2_Fe^II^FeIII2 (μ_3_-O)]^4+^ (the **S_1_** state). In the second step, the intensity of the MLCT band at 406 nm drastically decreased, suggesting that both iron centres at the apical positions are oxidised during the second step. Therefore, in the second step, the oxidation of the complex induces an intramolecular electron transfer process, and a species with two Fe^III^ ions at apical positions and one Fe^III^ and two Fe^II^ ions in the central core, [{Fe^III^(μ-bpp)_3_}_2_FeII2Fe^III^(μ_3_-O)]^5+^ (the **S_2_** state), forms. Further oxidation of the complex increased the intensity of the band at approximately 550 nm, which was attributed to the oxidation of the central core affording [{Fe^III^(μ-bpp)_3_}_2_Fe^II^FeIII2(μ_3_-O)]^6+^ (the **S_3_** state). Notably, these redox transformations of the complexes, which involve an intramolecular electron transfer process in the second step, are fully consistent with the previously reported Mössbauer study in the solid state.^33^,^64^ A similar trend was also observed for **[Fe_5_-Me]^3+^**, as shown in Fig. 7. Therefore, **[Fe_5_-H]^3+^** and **[Fe_5_-Me]^3+^** undergo an electron transfer reaction in an identical manner during their conversions from FeII4Fe^III^ to FeIII5 (Scheme 3, Path A), and the **S_4_** states serve as key intermediates in the catalytic reaction.

**Fig. 8:**
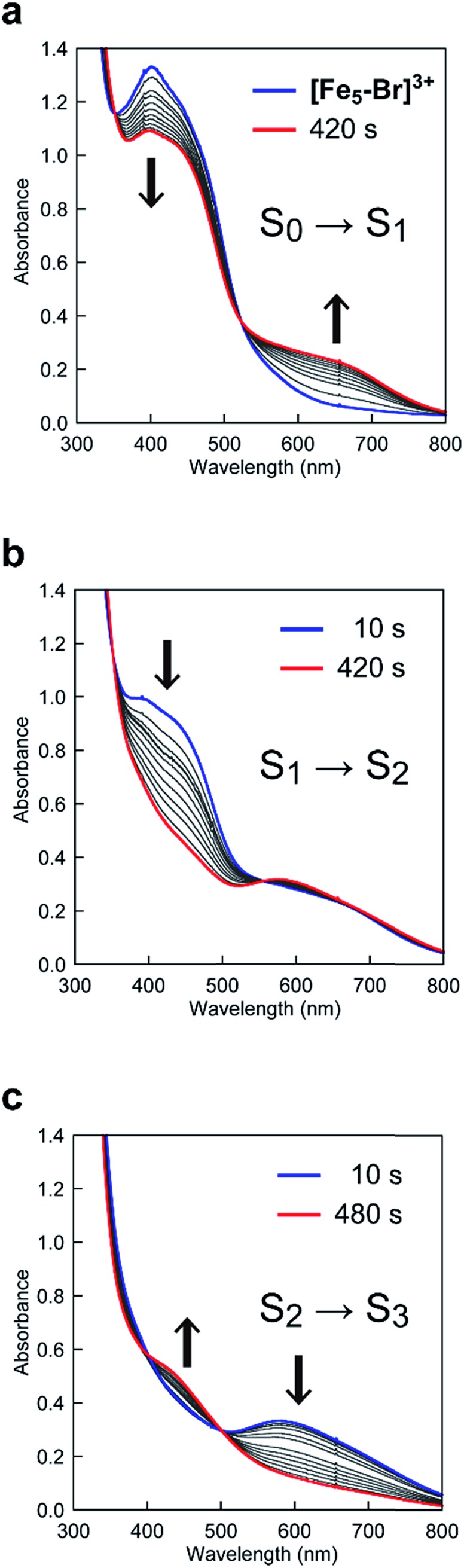
UV-Vis absorption spectra of **[Fe_5_-Br]^3+^** (0.05 mM) at various applied potentials ((a) 0.42, (b) 0.64 and (c) 1.00 V (*vs.* Fc/Fc^+^)) in 0.1 M TBAP/MeCN. Solutions were purged with Ar for 15 min prior to measurements. Weak Ar flow was maintained throughout the measurements.

In the case of **[Fe_5_-Br]^3+^**, the spectral changes that occurred upon the first and second oxidations were quite similar to those of **[Fe_5_-H]^3+^** and **[Fe_5_-Me]^3+^**; a slight decrease in the MLCT band at 402 nm and the emergence of a new band at approximately 660 nm in the first step and a drastic decrease in the MLCT band in the second step (Fig. 8a and b). Therefore, the iron ion at the central core is oxidised in the first step, and an oxidation-induced intramolecular electron transfer affords [{Fe^III^(μ-Br-bpp)_3_}_2_FeII2Fe^III^(μ_3_-O)]^5+^ (the **S_2_** state) in the second step. However, in the third step, the complex exhibited spectral changes that were unlike those of **[Fe_5_-H]^3+^** and **[Fe_5_-Me]^3+^**. In this step, the intensity of the band at approximately 580 nm decreased (Fig. 8c), whereas the corresponding bands for **[Fe_5_-H]^3+^** and **[Fe_5_-Me]^3+^** grew (*vide supra*). This result indicated that all iron atoms in the [Fe_3_(μ_3_-O)] core are oxidized in **[Fe_5_-Br]^6+^**. In other words, an additional intramolecular electron transfer reaction generates a three-electron oxidized species, and the oxidation states of the iron ions can be described as [{Fe^II^(μ-Br-bpp)_3_}{Fe^III^(μ-Br-bpp)_3_}FeIII3(μ_3_-O)]^6+^ (the **S_3_** state).

In the **[Fe_5_-Br]^3+^**-catalysed reaction, the formed **S_3_** state reacts with water *via* an electron transfer reaction coupled with the binding of H_2_O (*vide supra*). Thus, the oxidation of **[Fe_5_-Br]^3+^** to afford the key intermediate can be summarized as shown in Scheme 3, Path B. These results clearly demonstrate that the electronic structure of the **S_3_** state of **[Fe_5_-Br]^3+^** is completely different from that of **[Fe_5_-H]^3+^** and **[Fe_5_-Me]^3+^**, which may be the origin of the unique reactivity of **[Fe_5_-Br]^3+^** during catalysis.

### Reaction mechanism of **[Fe_5_-Br]^3+^**

**Fig. 9:**
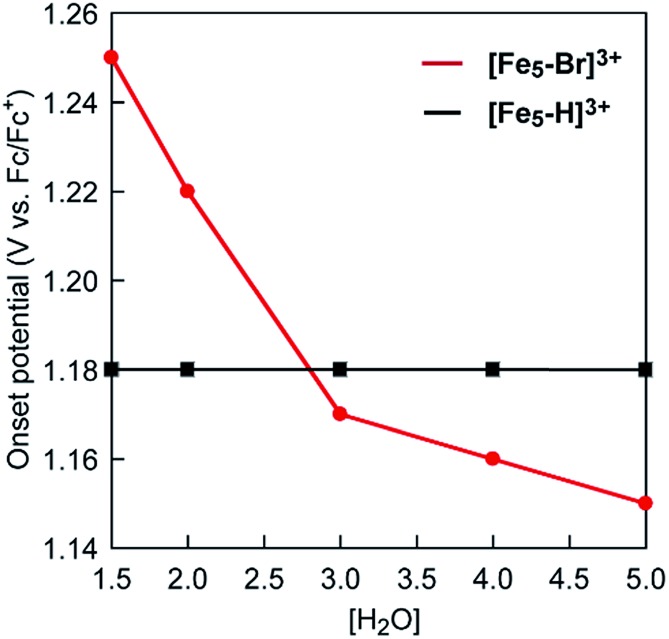
A plot of onset potentials as a function of the concentration of H_2_O for **[Fe_5_-Br]^3+^** (red line) and **[Fe_5_-H]^3+^** (black line).

Based on the aforementioned experimental evidence, a plausible reaction mechanism for the water oxidation reaction catalysed by **[Fe_5_-Br]^3+^** was proposed. As depicted in Scheme 3, the first step involves a sequential, stepwise three-electron oxidation of the initial **S_0_** state to produce the **S_3_** state (*via* the **S_1_** and **S_2_** states), which includes a two-step intramolecular electron transfer process. In the **S_3_** state of the complex, all iron atoms in the [Fe_3_(μ_3_-O)] core are in the Fe^III^ state. Subsequently, a concerted process involving water binding to the fully oxidized [Fe_3_(μ_3_-O)] core coupled with a one-electron oxidation process gives the water-bound FeIII5(OH_2_) species, intermediate **A** (Scheme 3, Path B). Intermediate **A** then generates intermediates **B** and **C**, and the release of O_2_ from intermediate **C** regenerates the initial **S_0_** state and produces O_2_ as a product (Fig. S19†). Thus, the formation of the **S_4_** state is favorably bypassed in the catalytic cycle of **[Fe_5_-Br]^3+^**, whereas the redox potentials to form the **S_4_** states determine the onset potentials for the catalysis in the case of **[Fe_5_-Me]^3+^** and **[Fe_5_-H]^3+^**. As a result, the onset potential for water oxidation was lower for **[Fe_5_-Br]^3+^** compared to **[Fe_5_-H]^3+^** if a sufficient amount of substrate was added to the reaction mixture (Fig. 9). The result also implies that the generation of the fully oxidized [Fe_3_(μ_3_-O)] core is essential for initiating catalysis.

## Conclusions

In conclusion, we have demonstrated the syntheses, electrochemical behaviour, and catalytic activity of a series of pentanuclear iron complexes. Two types of ligands bearing electron-donating (methyl) or electron-withdrawing (bromo) groups at the 4-position of the Hbpp ligand were successfully synthesized, and the complexation of the ligands (Me-Hbpp and Br-Hbpp) with iron resulted in the formation of pentanuclear complexes, **[Fe_5_-Me]^3+^** and **[Fe_5_-Br]^3+^**, which have structures similar to that of the parent complex, **[Fe_5_-H]^3+^**.

All three complexes exhibited five reversible redox waves, which can be attributed to the sequential redox processes of iron ions in the absence of H_2_O. However, the redox potentials of the complexes were significantly changed by the electronic effect of the substituents installed on the ligands. The redox potentials of **[Fe_5_-Me]^3+^** were shifted to more negative potentials compared to those of **[Fe_5_-H]^3+^**, whereas the redox waves of **[Fe_5_-Br]^3+^** were observed at more positive potentials than those of **[Fe_5_-H]^3+^**. In the presence of H_2_O, both **[Fe_5_-Me]^3+^** and **[Fe_5_-Br]^3+^** exhibited catalytic currents attributed to water oxidation reactions, similar to **[Fe_5_-H]^3+^**. CPE experiments of the complexes revealed that the faradaic efficiencies for the catalysis were 92 and 86% for **[Fe_5_-Me]^3+^** and **[Fe_5_-Br]^3+^**, respectively. The onset potentials for water oxidation by **[Fe_5_-Me]^3+^** and **[Fe_5_-Br]^3+^** were lower than that of **[Fe_5_-H]^3+^**.

To clarify the origin of the lower onset potentials of the complexes, their catalytic mechanisms were investigated. In the case of **[Fe_5_-Me]^3+^**, the formation of the **S_4_** state triggers the catalytic reaction, which is similar to the pathway seen with the parent **[Fe_5_-H]^3+^** complex. Therefore, the decrease in the onset overpotential for **[Fe_5_-Me]^3+^** is attributed to the electron-donating nature of the methyl substituents, which allows the generation of the **S_4_** state in the more negative potential region. In contrast, in the catalysis mediated by **[Fe_5_-Br]^3+^**, the three-electron oxidized species (the **S_3_** state) served as a key intermediate due to its unique electronic structure, and the state undergoes a water binding reaction coupled with an electron transfer to initiate the catalytic reaction. In other words, the generation of the **S_4_** state was bypassed in this system, which enables the catalytic reaction to occur at a lower onset potential. Our results reveal that not only the simple tuning of the redox potentials by the introduction of an electron-donating group but also the control over the reaction mechanism by the introduction of an electron-withdrawing group can be a valuable strategy for controlling onset potentials.

## Experimental section

### Materials

Pyridine-2-carboxylic acid methyl ester, 2-acetyl pyridine, 3,5-bis(2-pyridyl)pyrazole, methyl iodide, oxo[5,10,15,20-tetra(4-pyridyl)porphyrinato]titanium(iv), 1,8-diazabicyclo[5.4.0]undec-7-ene and tetraethylammonium perchlorate were purchased from Tokyo Chemical Industry Co., Ltd. NaBF_4_ and NaOH were purchased from Wako Pure Chemical Industries, Ltd. FeSO_4_·7H_2_O was purchased from Kanto Chemical Co., Inc. Potassium bis(trimethylsilyl)amide and tris(4-bromophenyl)ammoniumyl hexachloroantimonate were purchased from Aldrich and were used as received. An ITO-coated glass working electrode (0.7 mm thick, 10 Ω sq^–1^) was purchased from Furuuchi Chemical Co. Ltd. [FeII4Fe^III^(μ_3_-O)(bpp)_6_](BF_4_)_3_·7H_2_O ([Fe_5_-H](BF_4_)_3_·7H_2_O) was synthesized using the reported method.^33^ All solvents and reagents were of the highest quality available and were used as received except for tetrabutylammonium perchlorate (TBAP). Tetrabutylammonium perchlorate was recrystallized from absolute ethanol.

### General methods

NMR spectra were recorded on a JEOL JNM-LA 400 spectrometer. UV-Vis spectra were recorded on a SHIMADZU UV-2550UV-Vis spectrophotometer or a UV-Vis Agilent Cary8454 spectrophotometer with a conventional quartz cuvette (path length, *l* = 1 cm). Spectroelectrochemical studies were performed using a BAS Inc. spectroelectrochemical quartz cell (*l* = 1 mm) containing Pt gauze (working electrode), a Pt wire (auxiliary electrode) and Ag/Ag^+^ (reference electrode) in conjunction with a CH Instruments potentiostat. Elemental analyses were performed on a J-SCIENCE LAB MICRO CORDER JM10 elemental analyser. ESI-TOF mass spectra were recorded on a JEOL JMS-T100LP mass spectrometer. Gas chromatography analysis of O_2_ was performed using a Shimadzu GC-2014 gas chromatograph equipped with a thermal conductivity detector and fitted with a molecular sieve (5 Å) column, and the system was calibrated with air. Dynamic light scattering (DLS) data were measured using a Photal OTSUKA ELECTRONICS ELSZ-1000 zeta-potential and particle size analyser, equipped with a 785 nm red laser source (detection limit: 0.6 nm particle diameter).

### X-ray crystallography

Data collection for **[Fe_5_-Me]^3+^** and **[Fe_5_-Br]^3+^** was performed at 123 K on a ROD, Synergy Custom system (Rigaku Oxford Diffraction) equipped with confocal monochromated Mo-Kα radiation, and data were processed using CrysAlisPro 1.171.39.43c (Rigaku Oxford Diffraction). The structures were solved by direct methods using SIR-92 (ref. 66) and refined by the full-matrix least squares techniques on *F*^2^ (SHELXL-97).^67^ All nonhydrogen atoms were refined anisotropically and refined with a riding model with *U*_iso_ constrained to be 1.2 times *U*_eq_ of the carrier atom. The diffused electron densities resulting from the residual solvent molecules were removed from the data set using the SQUEEZE routine of PLATON^68^ and refined further using the generated data. Crystallographic data have been deposited with the Cambridge Crystallographic Data Centre: deposition numbers CCDC ; 1872481 and ; 1872482 for **[Fe_5_-Me]**(PF_6_)_3_ and **[Fe_5_-Br]**(BF_4_)_3_, respectively.

### Electrochemical studies

Electrochemical experiments were performed on a BAS ALS Model 650 DKMP electrochemical analyser at room temperature under Ar. Cyclic voltammetry experiments were performed using a one-compartment cell with a standard three-electrode configuration, which consisted of a glassy carbon disk (diameter 3 mm, from BAS Inc.), a Ag/Ag^+^ couple, and a platinum wire as the working, reference and auxiliary electrodes, respectively. Between scans, the working electrode was polished with 0.05 μm alumina paste (from BAS Inc.) and washed with purified H_2_O. All the redox potentials of the samples presented in this paper were calibrated against the redox potential of the ferrocene/ferrocenium couple (Fc/Fc^+^).

### Controlled potential electrolysis

Controlled potential electrolysis experiments were performed in a custom-designed gas-tight two-compartment cell separated by an anion-exchange membrane.^33^ In the first compartment, the ITO working electrode (1.0 cm × 1.5 cm) and Ag/Ag^+^ reference electrode were immersed in an electrolyte solution (0.1 M Bu_4_NClO_4_ in acetonitrile/water (10 : 1) mixed solvent) containing the catalyst (0.2 mM). In the second compartment, the platinum auxiliary electrode was immersed in the electrolyte solution. The amount of evolved oxygen in the headspace of the reaction cell was quantified by gas chromatography. Subsequently, the potential production of liquid products (*e.g.*, H_2_O_2_) in the reaction was analysed by treating the electrolyzed solution with oxo[5,10,15,20-tetra(4-pyridyl)porphyrinato]titanium(iv) as a chemical probe.^65^

### Syntheses

#### 1,3-Bis(2-pyridyl)-propane-1,3-dione (**I**)

To a solution of pyridine-2-carboxylic acid methyl ester (2 g, 16.5 mmol) in anhydrous toluene (40 mL) under an argon atmosphere was added freshly prepared sodium ethoxide solution (9.9 mL, 2 M, 19.8 mmol). After heating the reaction mixture to 55 °C, a solution of 2-acetyl pyridine (2.26 g, 16.5 mmol) in anhydrous toluene (10 mL) was added. After stirring the resulting mixture for 2 h at 55 °C, a dark yellow precipitate appeared, and the reaction was stirred overnight at room temperature. The solvent was then evaporated, and the crude product was poured onto ice and neutralized to pH 7 with acetic acid (50%). The resulting solid was collected by filtration and dried under vacuum to give compound **I** (1.92 g, 52%). The product was used for the next step without further purification.

#### 1,3-Bis(2-pyridyl)-propane-2-methyl-1,3-dione (**II**)

Under an argon atmosphere, compound **I** (0.5 g, 2.21 mmol) was dissolved in 30 mL of anhydrous toluene. Potassium bis(trimethylsilyl)amide (6.63 mL, 0.5 M, 3.32 mmol) was then added to the solution. The resulting suspension was heated to 80 °C, and the colour of the reaction mixture changed from orange to green. Then, methyl iodide (1.57 g, 11.05 mmol) was added. After heating the resulting mixture at 80 °C for 5 h, the reaction was stirred overnight at 50 °C. After cooling to room temperature, the reaction was quenched by the addition of 10% NaHCO_3_ (10 mL) followed by brine (10 mL) and extracted with CH_2_Cl_2_. The organic phases were dried over anhydrous Na_2_SO_4_ and filtered. The filtrate was concentrated under reduced pressure. The crude oily product was purified by silica gel column chromatography using 30% EtOAc/*n*-hexane as the eluent to give compound **II** as a light yellow solid (0.43 g, 82%). ^1^H NMR (400 MHz, CDCl_3_): *δ* = 8.48 (m, 2H), 8.07 (dt, *J* = 7.7 Hz, *J* = 1.2 Hz, 2H), 7.81 (td, *J* = 7.7 Hz, *J* = 1.7 Hz, 2H), 7.40 (m, 2H), 5.72 ppm (q, *J* = 7.06 Hz, 1H), 1.54, *J* = 7.02 Hz, 3H); ^13^C NMR (101 MHz, CDCl_3_): *δ* = 199.3, 152.4, 148.9, 137.2, 127.1, 122.5, 50.2, 13.2 ppm; elemental analyses calcd (%) for C_14_H_12_N_2_O_2_: C 69.99, H 5.03, N 11.66; found C 69.98, H 4.92, N 11.60.

#### 4-Methyl-3,5-bis(2-pyridyl)pyrazole (Me-Hbpp)

Compound **II** (0.33 g, 1.4 mmol) was dissolved in anhydrous ethanol (25 mL), and the solution was degassed with Ar for 30 min. To this solution was added hydrazine monohydrate (0.28 g, 5.6 mmol), and the reaction mixture was refluxed at 95 °C under Ar for 3 h. After concentrating the resulting solution by rotary evaporation, the solution was kept in a refrigerator overnight. A precipitate formed, and it was collected by filtration and washed with a small amount of cold ethanol to give Me-Hbpp as a white solid (0.19 g, 61%). ^1^H NMR (400 MHz, CDCl_3_): *δ* = 12.04 (s, 1H), 8.69 (m, 2H), 7.80 (d, *J* = 7.8 Hz, 2H), 7.78 (m, 2H), 7.25 (m, 2H), 2.74 ppm (s, 3H); ^13^C NMR (101 MHz, CDCl_3_): *δ* = 151.5, 149.5, 136.9, 122.5, 121.8, 113.8, 11.1 ppm; elemental analysis calcd (%) for C_14_H_12_N_4_: C 71.17, H 5.12, N 23.71; found C 70.74, H 5.13, N 23.43.

#### 4-Bromo-3,5-bis(2-pyridyl)-pyrazole (Br-Hbpp)

This compound was synthesized using the reported procedure.^63^ 3,5-Bis(2-pyridy)pyrazole (0.4 g, 1.8 mmol) was dissolved in CH_2_Cl_2_ (60 mL) at 0 °C. A solution of bromine (0.4 mL) in aqueous Na_2_CO_3_ (1 N, 25 mL) was then added dropwise, and the reaction was allowed to stir for 30 min. The reaction mixture was neutralized to pH 7 with aqueous 1 M NaOH. The aqueous phase was extracted with CH_2_Cl_2_ (80 mL). The organic phase was dried over anhydrous Na_2_SO_4_ and filtered. The filtrate was concentrated under reduced pressure to yield the crude product, which was purified by column chromatography on silica gel using 5% MeOH/CH_2_Cl_2_ to afford Br-Hbpp as a pale-yellow solid (0.35 g, 65%). ^1^H NMR (400 MHz, CD_2_Cl_2_): *δ* = 12.01 (br, 1H), 8.70 (m, 2H), 8.21 (d, *J* = 7.9 Hz, 2H), 7.84 (td, *J* = 7.9 Hz, *J* = 1.8 Hz), 7.34 (m, 2H); ^13^C NMR (101 MHz, CD_2_Cl_2_): *δ* = 149.4, 149.3, 144.9, 136.8, 123.4, 121.8, 91.1 ppm; elemental analysis calcd (%) for C_13_H_9_N_4_Br: C 51.59, H 3.01, N 18.60; found C 51.59, H 3.13, N 18.28.

#### [FeII4Fe^III^(μ_3_-O)(Me-bpp)_6_](PF_6_)_3_ ([Fe_5_-Me](PF_6_)_3_)

Me-Hbpp (0.040 g, 0.17 mmol) was dissolved in degassed methanol (10 mL), NaOH_aq_ (1 M, 0.17 mL, 0.17 mmol) was added, and the mixture was stirred to dissolve the Me-Hbpp. Subsequently, FeSO_4_·7H_2_O (0.038 g, 0.14 mmol) was added to the stirred solution, and the resulting dark red solution was refluxed at 80 °C for 1 h under Ar. After cooling the reaction mixture to room temperature, the mixture was filtered to remove the undissolved residue. An aqueous solution of NaPF_6_ (excess) was added to the filtrate, and a small amount of water was added to the solution. The solution was kept in a refrigerator for 30 min to generate a red brown precipitate. The precipitate was collected by filtration, washed with water and dried under vacuum. The obtained precipitate was dissolved in a mixture of acetonitrile and MeOH and subjected to vapour diffusion in diethyl ether to afford dark red crystals of [FeII4Fe^III^(μ_3_-O)(Me-bpp)_6_](PF_6_)_3_·5H_2_O. The crystals were collected by filtration and dried under vacuum. Yield 0.038 g (62%). Elemental analysis calcd (%) for Fe_5_C_84_H_76_N_24_P_3_F_18_O_6_: C 45.21, H 3.43, N 15.06; found C 45.22, H 3.16, N 15.08. ESI-TOF MS (positive ion, acetonitrile): *m*/*z*: 569.08 [FeII4Fe^III^(μ_3_-O)(Me-bpp)_6_]^3+^.

#### [FeII4Fe^III^(μ_3_-O)(Br-bpp)_6_](BF_4_)_3_ ([Fe_5_-Br](BF_4_)_3_)

A solution of FeSO_4_·7H_2_O (0.08 g, 0.28 mmol) in methanol (3 mL) was added to a stirred solution of Br-Hbpp (0.10 g, 0.33 mmol) and NaOH_aq_ (1 M, 0.33 mL, 0.33 mmol) in MeOH (10 mL). The resulting dark red solution was refluxed under air at 80 °C for 12 h. The reaction mixture was then filtered to remove the undissolved residue. The obtained filtrate was precipitated with a saturated aqueous solution of NaBF_4_ to give a brown precipitate, which was collected by filtration, washed with water, and dried under vacuum. The obtained precipitate was dissolved in acetonitrile and subjected to vapour diffusion in diethyl ether to afford dark red crystals of [FeII4Fe^III^(μ_3_-O)(Br-bpp)_6_](BF_4_)_3_·4H_2_O. The crystals were collected by filtration and dried under vacuum. Yield 0.06 g (43%). Elemental analysis calcd (%) for Fe_5_C_78_H_56_Br_6_N_24_B_3_F_12_O_5_: C 38.58, H 2.32, N 13.84; found C 38.79, H 2.45, N 13.77. ESI-TOF MS (positive ion, acetonitrile): *m*/*z*: 698.54 [FeII4Fe^III^(μ_3_-O)(Br-bpp)_6_]^3+^.

## Conflicts of interest

The authors declare no competing financial interests.

## Supplementary Material

Supplementary information

Crystal structure data
