# Supplementary material for: Pentanuclear iron catalysts for water oxidation: substituents provide two routes to control onset potentials
Source: Chem Sci. 2019 Mar 19;10(17):4628–39. doi: 10.1039/c9sc00678h (PMC6495723; doi:10.1039/c9sc00678h)
Supplement: Supplementary file 1 [file SC-010-C9SC00678H-s001.pdf]

Electronic Supplementary Material (ESI) for Chemical Science.
This journal is © The Royal Society of Chemistry 2019

# Electronic Supplementary Information

# Pentanuclear Iron Catalysts for Water Oxidation: Substituents Provide Two Routes to Control Onset Potentials

Vijayendran K. K. Praneeth,[a,†] Mio Kondo,[a,b,c,d,†] Masaya Okamura,[a] Takuya Akai,[a,b] Hitoshi Izu,[a,b] and Shigeyuki Masaoka*,[a,b,d]

[a.] Department of Life and Coordination-Complex Molecular Science, Institute for Molecular Science (IMS), 5-1 Higashiyama, Myodaiji, Okazaki, Aichi 444-8787, Japan.
[b.] SOKENDAI [The Graduate University for Advanced Studies], Shonan Village, Hayama, Kanagawa 240-0193, Japan.
[c.] ACT-C, Japan Science and Technology Agency (JST), 4-1-8 Honcho, Kawaguchi, Saitama 332-0012, Japan.
[d.] Research Center of Integrative Molecular Systems (CIMoS), Institute for Molecular Science (IMS), 38 Nishigo-naka, Myodaiji, Okazaki, Aichi 444-8585, Japan

E-mail: masaoka@ims.ac.jp, Fax: +81-564-59-5589

**Table of Contents**

**Syntheses** ........ S3
**Scheme S1.** ........ S3
**Scheme S2.** ........ S4
**Chart 1.** ........ S5
**Chart 2.** ........ S6
**Chart 3.** ........ S7
**Chart 4.** ........ S8
**Chart 5.** ........ S9
**Chart 6.** ........ S10
**X-ray Crystallography** ........ S11
**Table S1.** ........ S11
**Figure S1.** ........ S12
**Table S2.** ........ S13
**Figure S2.**. ........ S14
**UV-vis Absorption Spectroscopy** ........ S15
**Figure S3**. ........ S15
**Controlled Potential Electrolysis** ........ S16
**Figure S4.** ........ S16
**Figure S5.** ........ S17
**Figure S6.** ........ S18
**Figure S7.** ........ S19
**Figure S8.** ........ S20
**Figure S9.** ........ S21
**Detection Test for $H_2O_2$** ........ S22
**Figure S10.** ........ S23
**Figure S11.** ........ S23
**Estimation of TOF and TON from Controlled Potential Electrolysis** ... S24
**Electrochemical Measurements** ........ S25
**Figure S12.** ........ S25
**Figure S13.** ........ S26
**Figure S14.** ........ S27
**Figure S15.** ........ S28
**Figure S16.** ........ S29
**Figure S17.** ........ S30
**Figure S18.** ........ S31
**Reaction Mechanism** ........ S32
**1. Reaction mechanism of $[Fe_5\text{-}H]^{3+}$ and $[Fe_5\text{-}Me]^{3+}$** ........ S32
**2. Reaction mechanism of $[Fe_5\text{-}Br]^{3+}$** ........ S33
**Figure S19.**. ........ S33
**References** ........ S34
**Author Contributions** ........ S34

## Syntheses

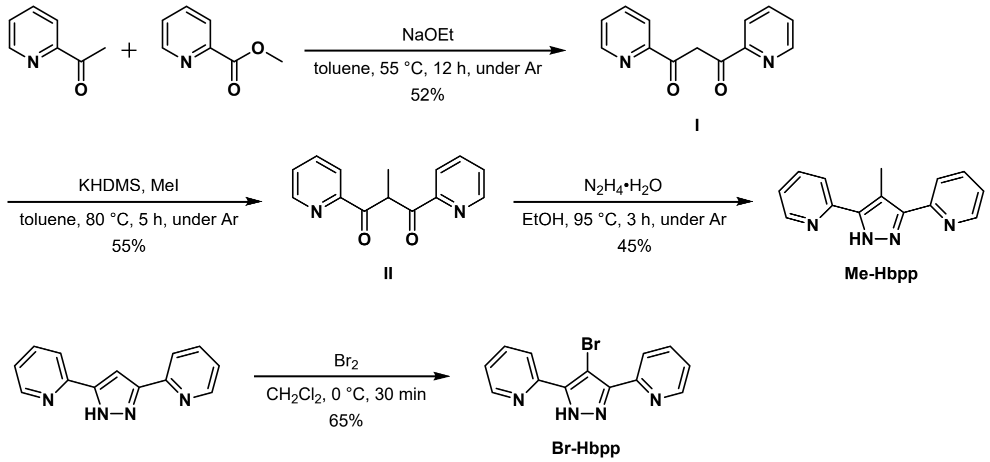


**Scheme S1.** Syntheses of Me-Hbpp and Br-Hbpp

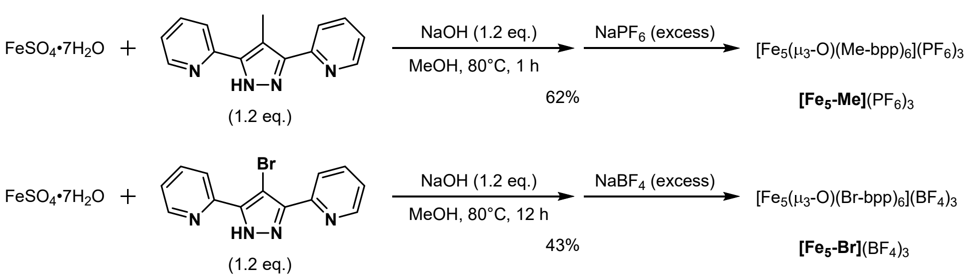


**Scheme S2.** Syntheses of **[Fe$_5$-Me]**$^{3+}$ and **[Fe$_5$-Br]**$^{3+}$.

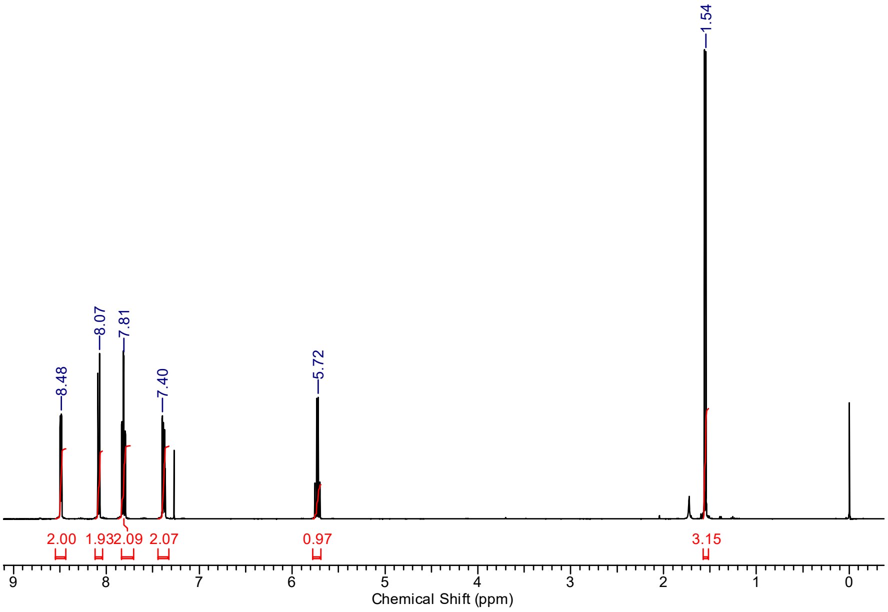


**Chart 1.** $^{1}$H NMR spectrum of 1,3-Bis(2-pyridyl)-propane-2-methyl-1,3-dione (II) in $CDCl_3$ at 296 K.

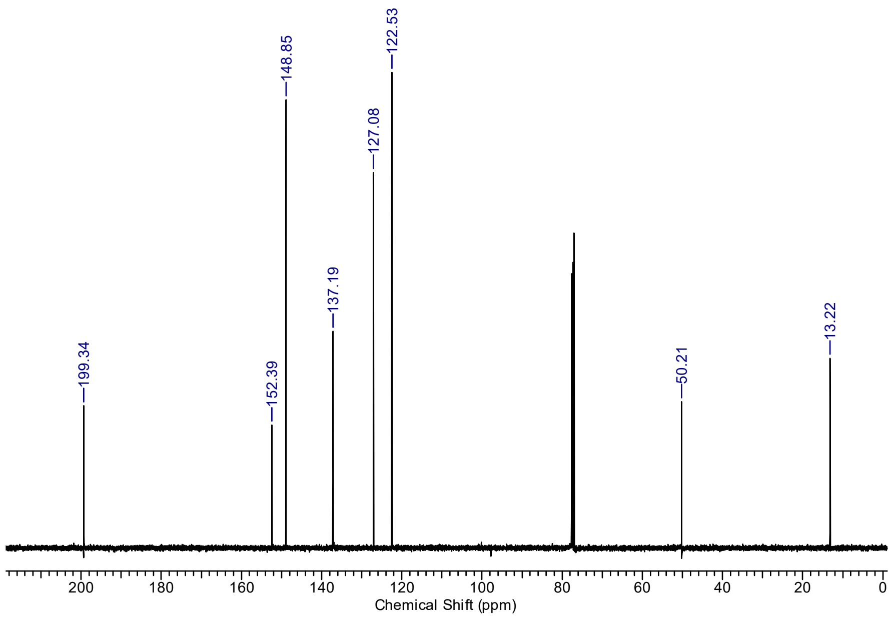


**Chart 2.** $^{13}C$ NMR spectrum of 1,3-bis(2-pyridyl)-propane-2-methyl-1,3-dione (II) in $CDCl_3$ at 296 K.

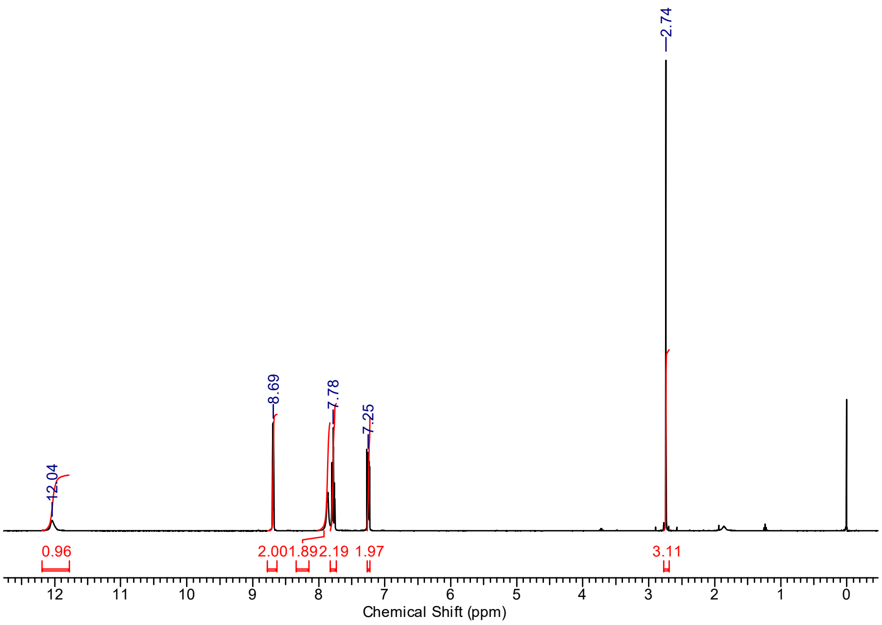


**Chart 3.** $^{1}H$ NMR spectrum of 4-methyl-3,5-bis(2-pyridyl)pyrazole (Me-Hbpp) in $CDCl_3$ at 296 K.

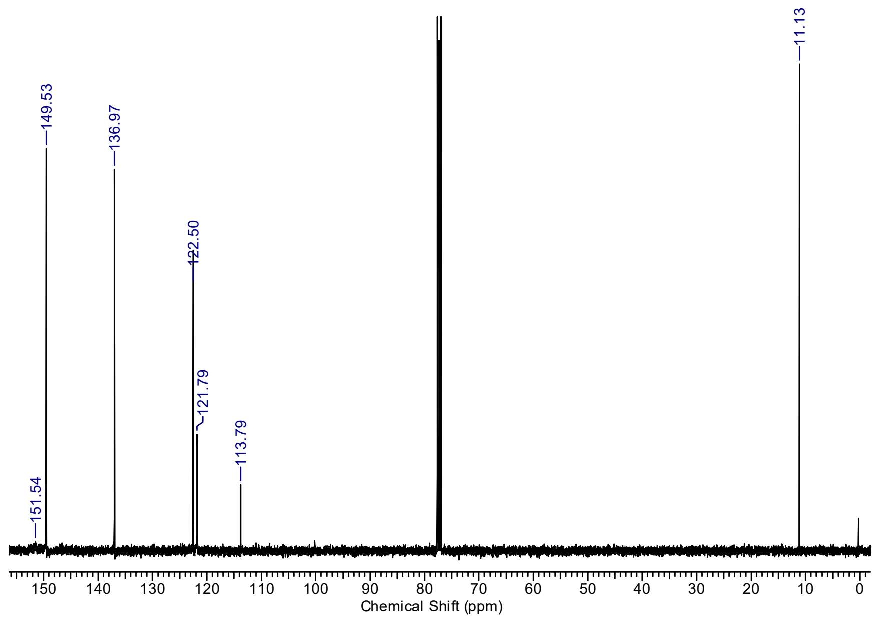


**Chart 4.** $^{13}C$ NMR spectrum of 4-methyl-3,5-bis(2-pyridyl)pyrazole (Me-Hbpp) in $CDCl_3$ at 296 K.

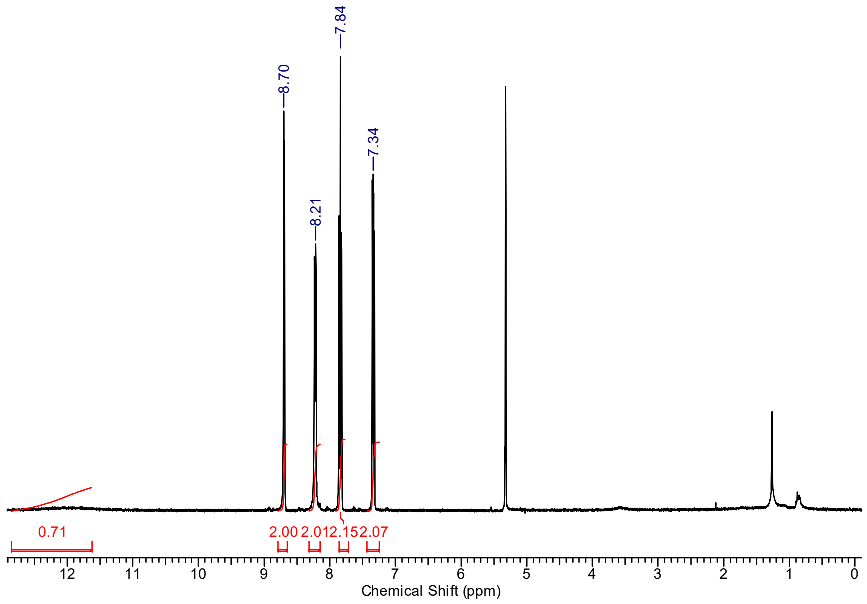


**Chart 5.** $^{1}$H NMR spectrum of 4-bromo-3,5-bis(2-pyridyl)pyrazole (Br-Hbpp) in $CD_2Cl_2$ at 296 K.

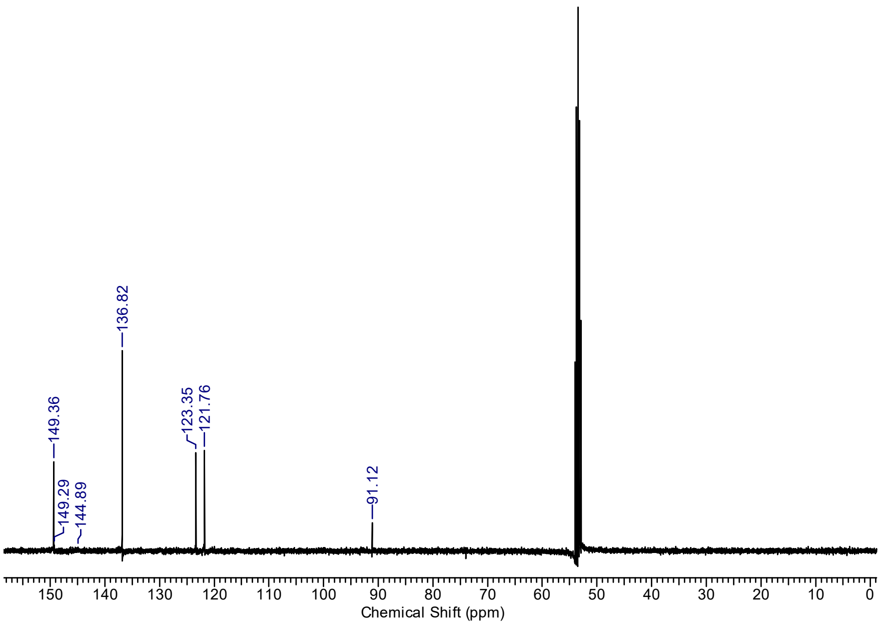


**Chart 6.** $^{13}C$ NMR spectrum of 4-bromo-3,5-bis(2-pyridyl)pyrazole (Br-Hbpp) in $CD_2Cl_2$ at 296 K.

## X-ray Crystallography

**Table S1.** Summary of crystallographic data for **[Fe$_5$-Me]**$(PF_6)_3$, **[Fe$_5$-Br]**$(BF_4)_3$, and **[Fe$_5$-H]**$(BF_4)_3$.

| Complex | **[Fe$_5$-Me]**$(PF_6)_3$ | **[Fe$_5$-Br]**$(BF_4)_3$ | **[Fe$_5$-H]**$(BF_4)_3$[S1] |
|---|---|---|---|
| Formula | $C_{84}H_{66}N_{24}OFe_5P_3F_{18}$ | $C_{78}H_{48}N_{24}OBr_6Fe_5B_3F_{12}$ | $C_{78}H_{54}N_{24}OFe_5B_3F_{12}$ |
| Fw | 2141.76 | 2356.54 | 1883.13 |
| crystal color, | dark orange, plate | clear dark black, plate | red, block |
| crystal size, mm$^3$ | 0.76 × 0.51 × 0.28 | 0.59 × 0.34 × 0.10 | 0.22 × 0.1 × 0.08 |
| crystal system | triclinic | trigonal | tetragonal |
| space group | $P\overline{1}$ | $R\overline{3}$ | $I\overline{4}$ |
| $a$ / Å | 18.1532(3) | 19.1174(5) | 16.6876(3) |
| $b$ / Å | 18.1744(3) | 19.1174(5) | 16.6876(3) |
| $c$ / Å | 18.5745(4) | 45.8919(14) | 30.1763(7) |
| α / deg | 70.728(2) | 90 | 90 |
| β / deg | 65.127(2) | 90 | 91.143(2) |
| γ / deg | 79.662(2) | 120 | 90 |
| $V$ / Å$^3$ | 5242.3(2) | 14525.3(9) | 8403.4(3) |
| $Z$ | 2 | 6 | 4 |
| $F$(000) | 2166 | 6966 | 3804 |
| $d_{calc}$, g/cm$^3$ | 1.357 | 1.621 | 1.488 |
| $\mu$(MoK$\alpha$), mm$^{-1}$ | 0.806 | 3.285 | 0.929 |
| $T$ / K | 123(2) | 123(2) | 123(2) |
| $R_1$ | 0.0430 | 0.0536 | 0.0526 |
| w$R_2$ | 0.1219 | 0.1467 | 0.1503 |
| GOF | 1.020 | 1.033 | 1.099 |

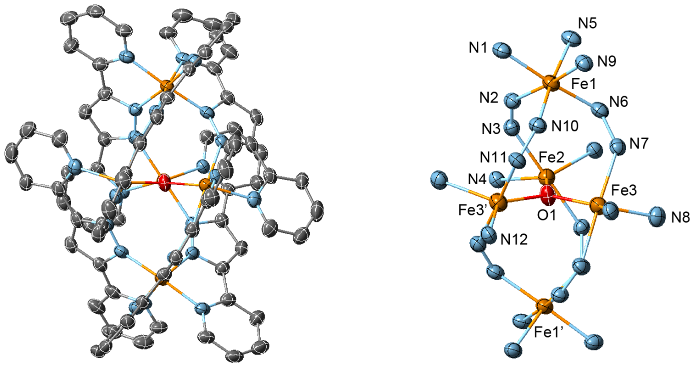


**Figure S1.** ORTEP drawings of (top) the cationic moiety and (bottom) the core structure of **[Fe$_5$-H]**$(BF_4)_3$.[S1] The atoms are represented by the following colours: Fe, orange; O, red; N, blue; C, grey; and Br, purple. Hydrogen atoms and crystal solvent molecules are omitted for clarity. Thermal ellipsoids are shown at 50% probability.

**Table S2.** Selected bond lengths and angles of **[Fe$_5$-Me]**$(PF_6)_3$, **[Fe$_5$-Br]**$(BF_4)_3$ and **[Fe$_5$-H]**$(BF_4)_3$

| | [Fe$_5$-Me]$^{3+}$ | | [Fe$_5$-Br]$^{3+}$ | | [Fe$_5$-H]$^{3+}$[S1] | |
|---|---|---|---|---|---|---|
| apical | Fe1-N1 | 1.996(2) | Fe1-N1 | 1.994(3) | Fe1-N1 | 2.017(3) |
| | Fe1-N2 | 1.9636(18) | Fe1-N2 | 1.951(3) | Fe1-N2 | 1.966(3) |
| | Fe1-N5 | 2.005(2) | Fe2-N5 | 1.993(7) | Fe1-N5 | 2.018(3) |
| | Fe1-N6 | 1.9591(19) | Fe2-N6 | 1.955(5) | Fe1-N6 | 1.971(3) |
| | Fe1-N9 | 2.0002(18) | | | Fe1-N9 | 2.004(3) |
| | Fe1-N10 | 1.960(2) | | | Fe1-N10 | 1.963(3) |
| | Fe2-N13 | 1.983(2) | | | | |
| | Fe2-N14 | 1.9660(19) | | | | |
| | Fe2-N17 | 2.002(2) | | | | |
| | Fe2-N18 | 1.9533(18) | | | | |
| | Fe2-N21 | 1.9975(19) | | | | |
| | Fe2-N22 | 1.956(2) | | | | |
| **average** | | **1.9785(19)** | | **1.973(5)** | | **1.990(3)** |

| | [Fe$_5$-Me]$^{3+}$ | | [Fe$_5$-Br]$^{3+}$ | | [Fe$_5$-H]$^{3+}$ | |
|---|---|---|---|---|---|---|
| core-N | Fe3-N3 | 2.097(2) | Fe3-N3 | 2.116(3) | Fe2-N3 | 2.085(3) |
| | Fe3-N4 | 2.1016(19) | Fe3-N4 | 2.106(3) | Fe2-N4 | 2.141(3) |
| | Fe3-N15 | 2.098(2) | Fe3-N7 | 2.100(4) | Fe3-N7 | 2.089(3) |
| | Fe3-N16 | 2.1009(19) | Fe3-N8 | 2.116(4) | Fe3-N8 | 2.118(3) |
| | Fe4-N7 | 2.1035(19) | | | Fe3-N11 | 2.127(3) |
| | Fe4-N8 | 2.1199(19) | | | Fe3-N12 | 2.106(3) |
| | Fe4-N19 | 2.1014(19) | | | | |
| | Fe4-N20 | 2.1198(18) | | | | |
| | Fe5-N11 | 2.1106(18) | | | | |
| | Fe5-N12 | 2.1059(19) | | | | |
| | Fe5-N3 | 2.1101(18) | | | | |
| | Fe5-N24 | 2.117(2) | | | | |
| **average** | | **2.1071(19)** | | **2.110(4)** | | **2.111(3)** |

| | [Fe$_5$-Me]$^{3+}$ | | [Fe$_5$-Br]$^{3+}$ | | [Fe$_5$-H]$^{3+}$ | |
|---|---|---|---|---|---|---|
| core-O | Fe3-O1 | 1.8551(15) | Fe3-O1 | 1.8734(6) | Fe2-O1 | 1.983(4) |
| | Fe4-O1 | 1.8812(15) | | | Fe3-O1 | 1.9183(19) |
| | Fe5-O1 | 1.8708(15) | | | | |
| **average** | | **1.8690(15)** | | **1.8734(6)** | | **1.961(3)** |
| Core angle | Fe3-O1-Fe4 | 119.09(8) | Fe3-O1-Fe3' | 119.985(7) | Fe2-O1-Fe3 | 120.69(9) |
| | Fe3-O1-Fe5 | 119.67(8) | | | Fe3-O1-Fe3' | 118.63(18) |
| | Fe4-O1-Fe5 | 121.23(8) | | | | |
| **average** | | **120.00(8)** | | **119.985(7)** | | **120.0(1)** |

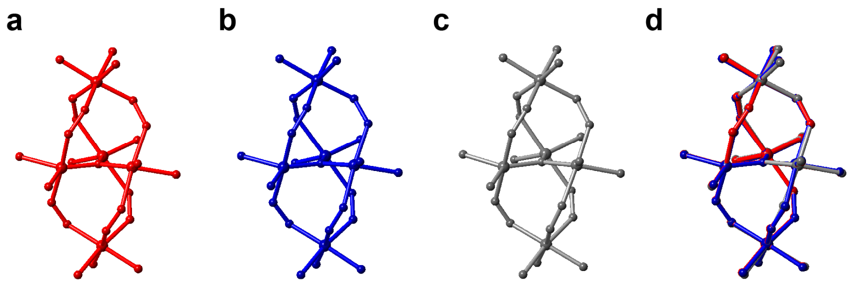


**Figure S2.** Comparison of core structures of (a) **[$Fe_5$-Me]**($PF_6$)$_3$, (b) **[$Fe_5$-Br]**($BF_4$)$_3$, and (c) **[$Fe_5$-H]**($BF_4$)$_3$[S1] and (d) an overlaid image of the core structures of the three complexes. **[$Fe_5$-Me]**(TfO)$_3$, **[$Fe_5$-Br]**($BF_4$)$_3$, and **[$Fe_5$-H]**($BF_4$)$_3$ are drawn in red, blue and grey colour, respectively.

## UV-vis Absorption Spectroscopy

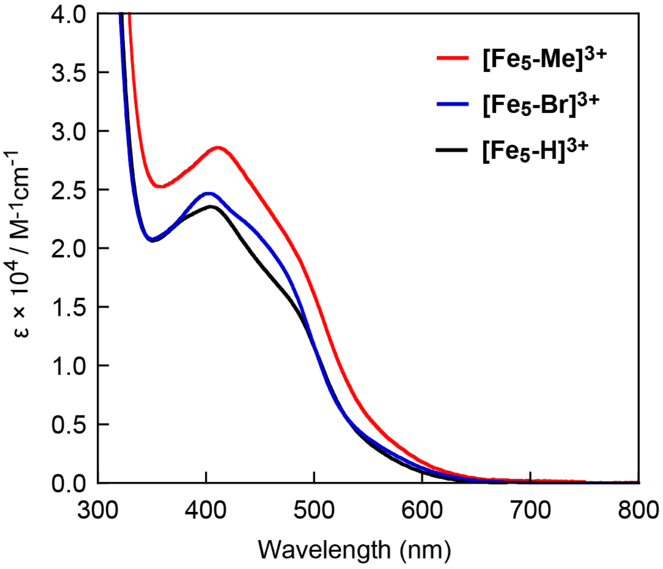


**Figure S3**. UV-vis absorption spectra of **[Fe$_5$-Me]$^{3+}$**, **[Fe$_5$-Br]$^{3+}$** and **[Fe$_5$-H]$^{3+}$** in MeCN under Ar at 293 K.

## Controlled Potential Electrolysis

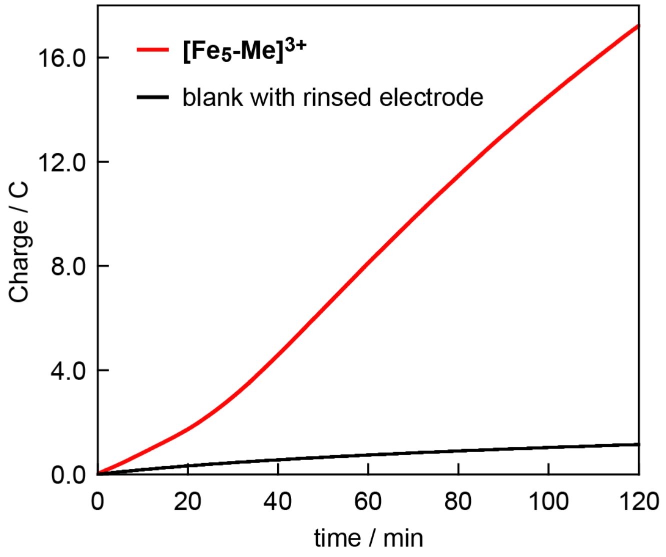


**Figure S4.** The result of the first electrolysis using fresh ITO electrode in 0.2 mM of **[Fe$_5$-Me]$^{3+}$** (red line) and that of second round of electrolysis using ITO electrode after first electrolysis in electrolyte solution without **[Fe$_5$-Me]$^{3+}$** (black line). Condition: MeCN containing 0.1 M TBAP with added $H_2O$ (5 M) at a potential of 1.42 V *vs.* Fc/Fc$^+$.

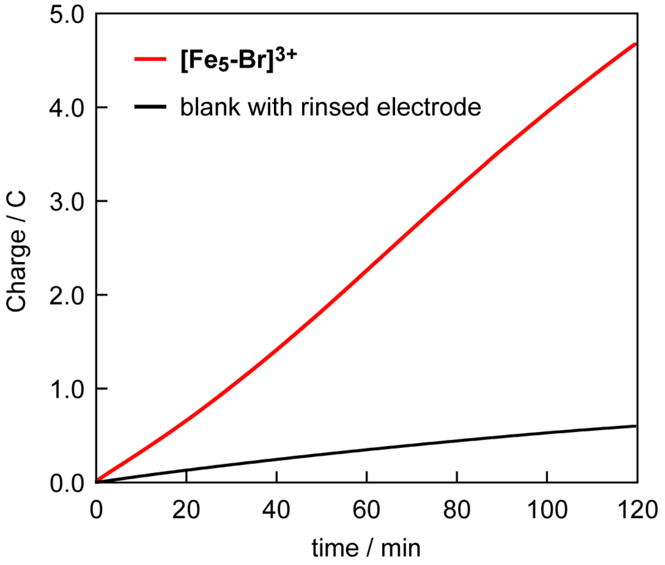


**Figure S5.** The result of the first electrolysis using fresh ITO electrode in 0.2 mM of **[$Fe_5$-Br]$^{3+}$** (red line) and that of second round of electrolysis using ITO electrode after first electrolysis in electrolyte solution without **[$Fe_5$-Br]$^{3+}$** (black line). Condition: MeCN containing 0.1 M TBAP with added $H_2O$ (5 M) at a potential of 1.42 V *vs.* Fc/Fc$^+$.

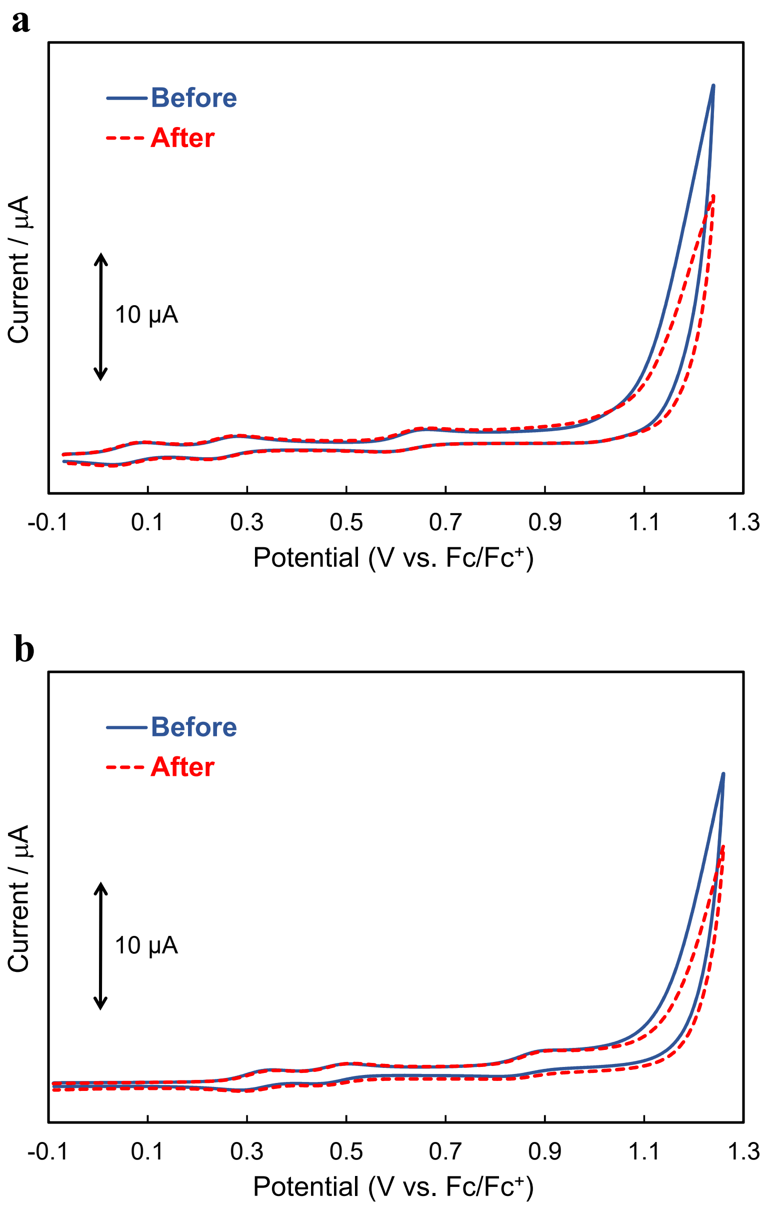


**Figure S6.** CVs obtained before (blue lines) and after (red lines) the controlled potential electrolysis of (a) **[$Fe_5$-Me]$^{3+}$** and (b) **[$Fe_5$-Br]$^{3+}$**. The CV measurements were performed in acetonitrile/water (10 : 1) mixed solution with 0.1 M TBAP as the supporting electrolyte at a scan rate of 10 mV $s^{-1}$. The controlled potential electrolysis were performed at 1.24 V vs. Fc/$Fc^+$ with 0.2 mM solutions of **[$Fe_5$-Me]$^{3+}$** or **[$Fe_5$-Br]$^{3+}$** for 1 h at pH = 5.0. After the electrode was polished with alumina paste, the CVs were recorded (red lines). Both the wave shapes of the Fe(II/III) processes and the catalytic current for water oxidation were maintained after the controlled potential electrolysis in both the cases. Electrodes: working, GC; auxiliary, Pt; reference, Ag/$Ag^+$; reported vs. Fc/$Fc^+$.

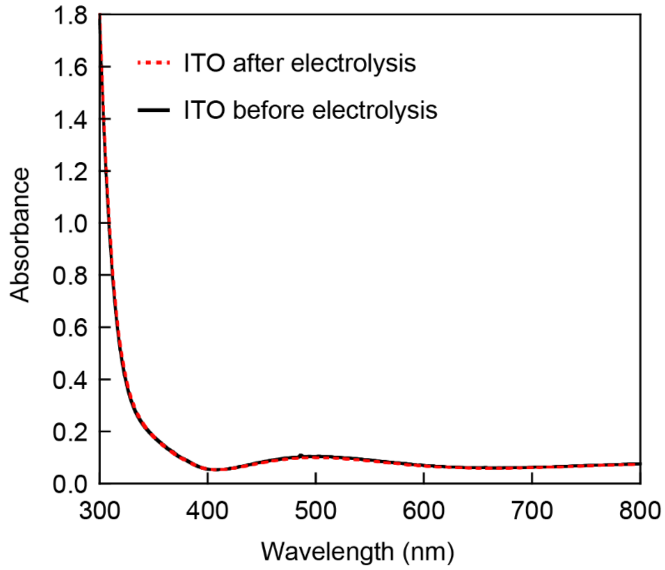


**Figure S7.** UV-vis absorption spectra of the ITO electrode before (black line) and after (red dotted line) the electrolysis of **[Fe$_5$-Me]**$^{3+}$.

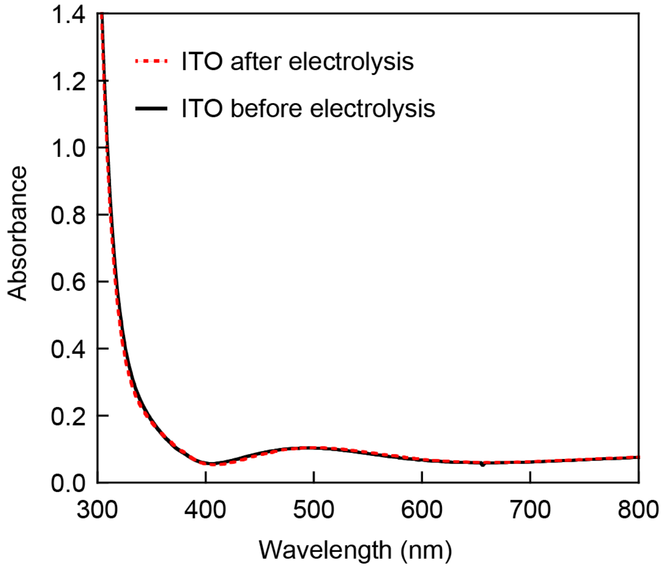


**Figure S8.** UV-vis absorption spectra of the ITO electrode before (black line) and after (red dotted line) the electrolysis of **$[Fe_5\text{-}Br]^{3+}$**.

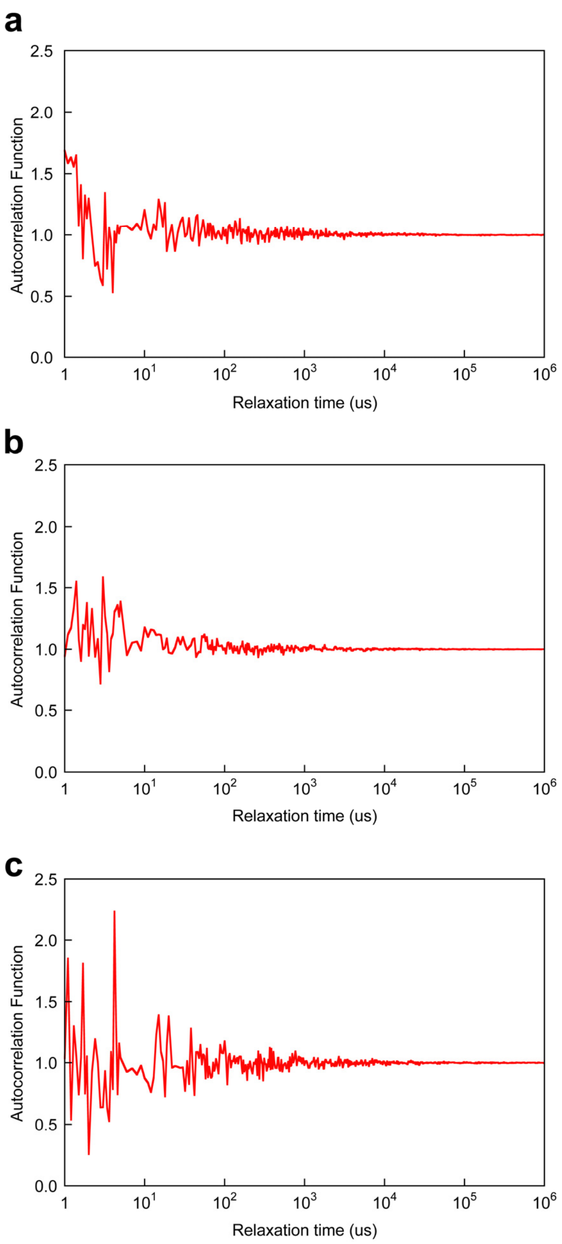


**Figure S9.** Autocorrelation functions obtained by DLS measurements of electrolyte solutions after the CPE experiments of (a) **[Fe$_5$-Me]$^{3+}$** and (b) **[Fe$_5$-Br]$^{3+}$**, and the blank solution.

## Detection Test for $H_2O_2$

The electrolyzed solutions of **[$Fe_5$-Me]$^{3+}$** and **[$Fe_5$-Br]$^{3+}$** were treated with the water-soluble titanium(IV)porphyrin complex, oxo[5,10,15,20-tetra(4-pyridyl)porphyrinato]titanium (IV), as a chemical probe, in order to detect any $H_2O_2$ (2e$^-$ oxidized product of $H_2O$) formation. The procedure was followed according to the literature method by Inoue *et al.* (*ChemSusChem*, **2017**, *10*, 1909). An aqueous standard solution of oxo[5,10,15,20-tetra(4-pyridyl)porphyrinato]titatium (IV) ($3 \times 10^{-5}$ M) was first prepared in 1 M $H_2SO_4$. The reaction mixtures after the controlled potential electrolysis experiments of **[$Fe_5$-Me]$^{3+}$** and **[$Fe_5$-Br]$^{3+}$** were passed through a short silica gel column in order to remove the iron complexes from the solutions. **[$Fe_5$-Me]$^{3+}$** and **[$Fe_5$-Br]$^{3+}$** were effectively adsorbed on the silica gel and were removed from the solutions. The decolorized solutions (2 mL from each of the solution) were then mixed with 1 mL of the standard solution and stirred in the dark for 30 min. The UV-Vis absorption spectrum of the probe was measured before and after its treatment with the electrolyzed solutions. The presence of $H_2O_2$ can be determined quantitatively from the absorption spectral changes (a decrease of the absorption band at $\lambda$ = 432 nm, accompanied by an increase in the absorption of a new band at $\lambda$ = 445 nm). However, after the treatment of the electrolyzed solutions of **[$Fe_5$-Me]$^{3+}$** and **[$Fe_5$-Br]$^{3+}$** with the titanium(IV)porphyrin complex showed no such spectral changes (Figures S10 and S11). This indicates no $H_2O_2$ formation in the **[$Fe_5$-Me]$^{3+}$** and **[$Fe_5$-Br]$^{3+}$**-catalysed reactions.

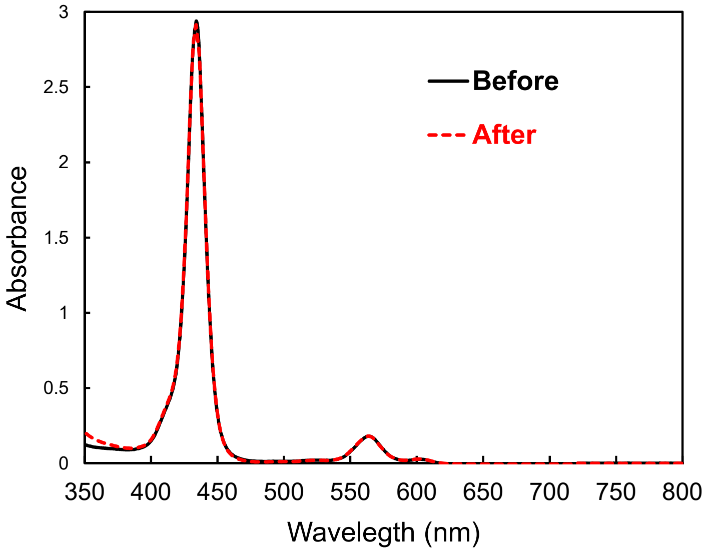


**Figure S10.** UV-Vis absorption spectra of oxo[5,10,15,20-tetra(4-pyridyl)porphyrinato]titanium (IV), before (black line) and after (red line) treatment with the electrolyzed solution of **[Fe$_5$-Me]**$^{3+}$.

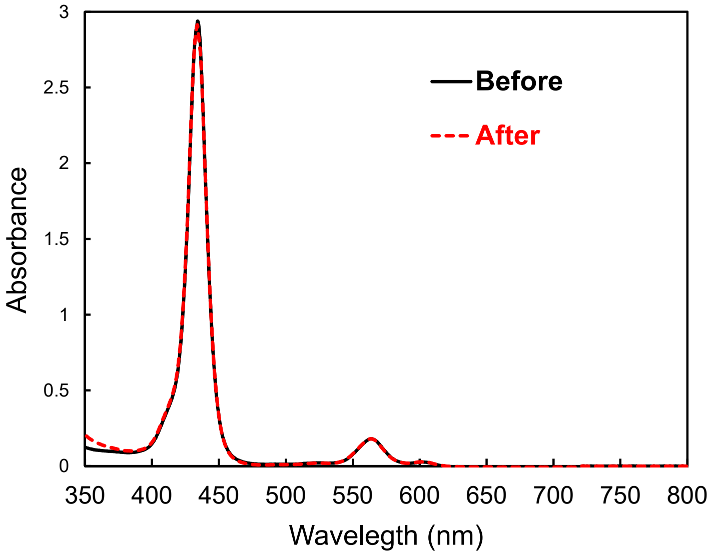


**Figure S11.** UV-Vis absorption spectra of oxo[5,10,15,20-tetra(4-pyridyl)porphyrinato]titanium (IV) before (black line) and after (red line) treatment with the electrolyzed solution of **[Fe$_5$-Br]**$^{3+}$.

## Estimation of TOF and TON from Controlled Potential Electrolysis

The TOF and TON values from the CPE data were determined based on the previously reported analysis (Equations 1-4) by Costentin and Savéant (*J. Am. Chem. Soc.* **2012**, *134*, 11235-11242).

$$\frac{i}{F\ \mathrm{A}} = \frac{\sqrt{k_{cat}\ D}\ [\mathrm{cat}]}{1+\exp\left[-\ \frac{F}{RT}\ (E_{applied}-E^{0}_{\mathrm{cat}})\right]} \qquad \text{(Eq. 1)}$$

$$k_{\mathrm{cat}} = \frac{i^{2}\left(1+exp\left[-\ \frac{F}{RT}\ (E_{applied}-E^{0}_{\mathrm{cat}})\right]\right)^{2}}{F^{2}\ \mathrm{A}^{2}\ D\ [cat]^{2}} \qquad \text{(Eq. 2)}$$

$$\mathrm{TOF} = \frac{k_{cat}}{1+\exp\left[-\ \frac{F}{RT}\ (E_{applied}-E^{0}_{\mathrm{cat}})\right]} \qquad \text{(Eq. 3)}$$

$$\mathrm{TON} = \mathrm{TOF} \times \mathrm{time} \qquad \text{(Eq. 4)}$$

The equations were adapted and used in the water oxidation reaction previously by Lin *et al.* (*J. Am. Chem. Soc.* **2014**, *136*, 273-281). In these above equations, *i* represents the current transferred during CPE, *F* is Faraday's constant (96 485 C/mol), A is the surface area of the ITO electrode (3 $cm^2$), $k_{cat}$ is the overall rate constant of the catalytic water oxidation reaction, *D* is the diffusion coefficient, which is assumed to be ~5 × $10^{-6}$ $cm^2/s$, [cat] is the initial concentration of the catalysts (0.2 mM = 2 × $10^{-7}$ mol/$cm^3$, *R* is the universal gas constant (8.31 J $K^{-1}$ $mol^{-1}$), *T* is temperature (298.15 K), $E_{applied}$ is the applied potential during CPE (1.42 V vs. Fc/$Fc^+$), $E^0_{cat}$ is the standard potential of the catalyst ($E_{cat/2}$ values were used: 1.19 V vs. Fc/$Fc^+$ for **$[Fe_5\text{-}Me]^{3+}$** and 1.26 V vs. Fc/$Fc^+$ for **$[Fe_5\text{-}Br]^{3+}$**), and TOF is the turnover frequency. This leads to a calculated value of TOF = 3 × $10^2$ $s^{-1}$ and TON = 2 × $10^6$ for **$[Fe_5\text{-}Me]^{3+}$** and TOF = 20 $s^{-1}$ and TON = 1 × $10^5$ for **$[Fe_5\text{-}Br]^{3+}$**. We also determined the TOF and TON values of **$[Fe_5\text{-}H]^{3+}$** using the equation described above (Eqs 1-4), and values were determined to be 1 × $10^3$ $s^{-1}$ (TOF) and 7.5 × $10^6$ (TON). These values were comparable to those we reported in the previous study[S1], although the method to obtain the values were slightly different.

**Electrochemical Measurements**

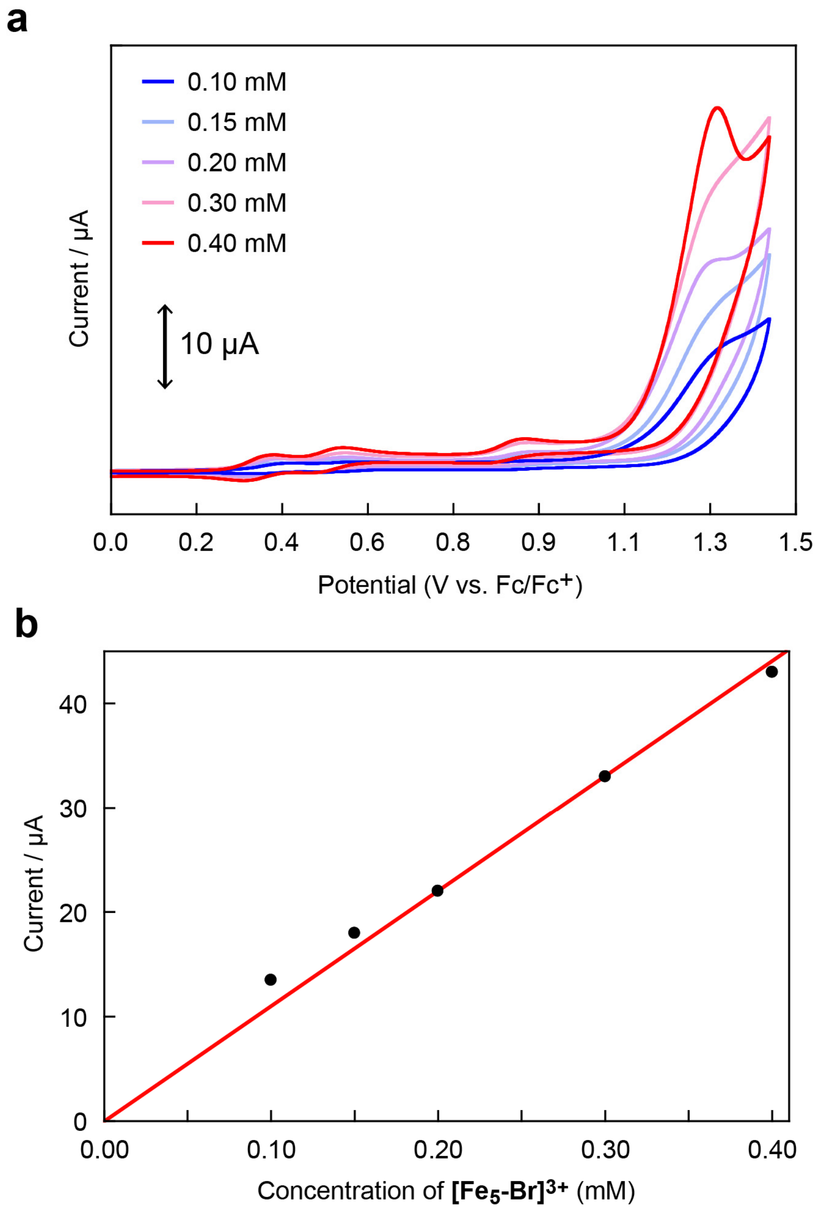


**Figure S12.** (a) Cyclic voltammograms of **[Fe₅-Br]$^{3+}$** at various concentrations in the presence of 5 M $H_2O$ (pH = 5) in MeCN with TBAP (0.1 M) at a scan rate of 10 mV $s^{-1}$, under Ar. (b) A plot of catalytic peak current ($i_{cat}$) *vs.* concentrations of **[Fe$_5$-Br]$^{3+}$** at 1.3V vs. Fc/Fc$^+$.

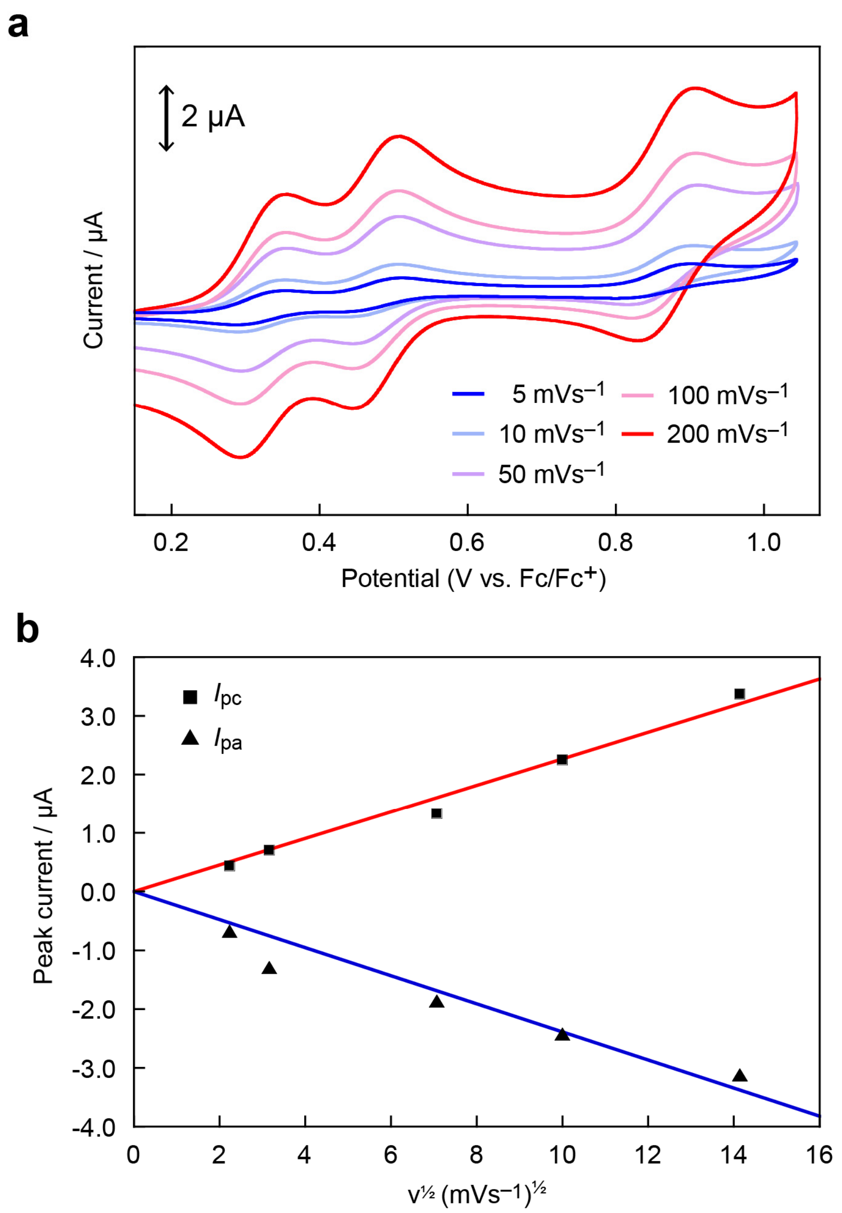


**Figure S13.** (a) Cyclic voltammograms of [**Fe$_5$-Br**]$^{3+}$ (0.2 mM) in the presence of 5 M $H_2O$ (pH = 5) under varying scan rates with the applied switching potential of the reverse scan at 1.04 V. (b) A plot of anodic ($I_{pa}$) and cathodic ($I_{pc}$) peak currents of the 3$^{rd}$ redox couple versus square root of the scan rates. CVs were measured in acetonitrile solutions with TBAP (0.1 M).

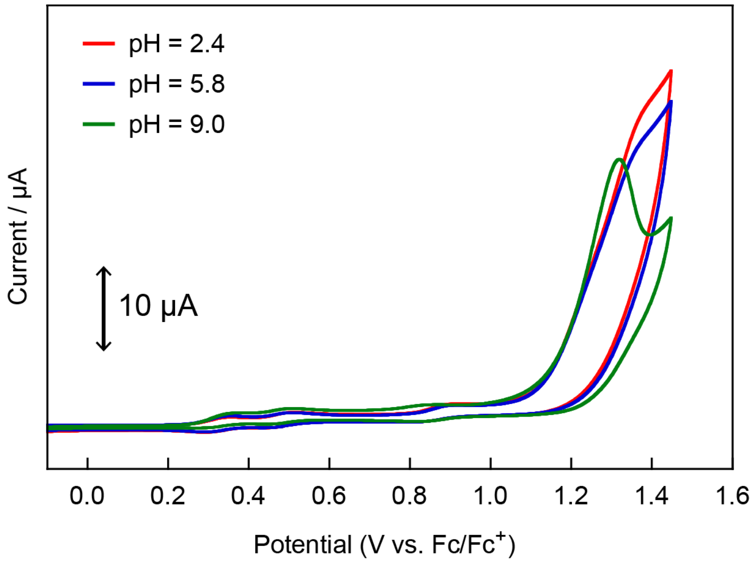


**Figure S14.** Cyclic voltammograms of **[Fe$_5$-Br]**$^{3+}$ (0.2 mM) in acetonitrile solutions with TBAP (0.1 M) at a scan rate of 10 mV s$^{-1}$, in the presence of 5 M of $H_2O$ at various pH conditions. The CVs at various pH showed no change of the onset potential of water oxidation.

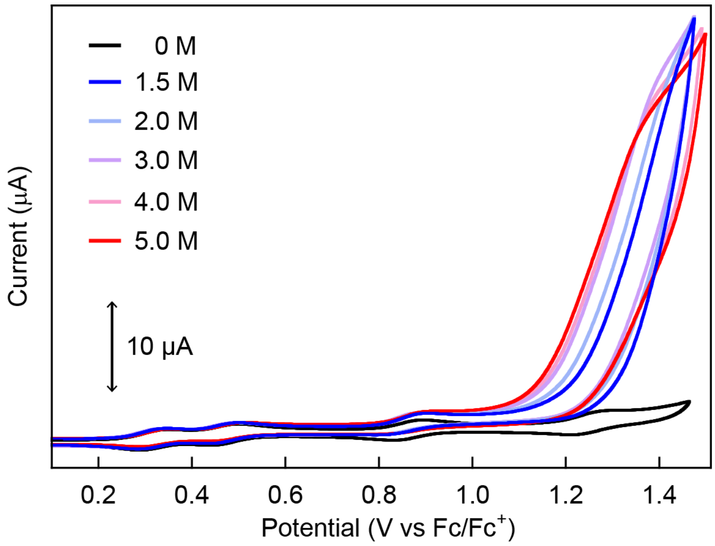


**Figure S15.** Cyclic voltammograms of **[Fe$_5$-Br]$^{3+}$** (0.2 mM) in the presence of (a) 1.5, (b) 2.0, (c) 3.0, (d) 4.0, (e) 5.0 M of $H_2O$ (pH = 5, red lines) and in the absence of $H_2O$ (black lines). (f) Overlaid cyclic voltammograms of **[Fe$_5$-Br]$^{3+}$** at various concentrations of $H_2O$. Cyclic voltammograms are measured in acetonitrile solutions with TBAP (0.1 M) on a GC electrode at a scan rate of 10 mV s$^{-1}$.

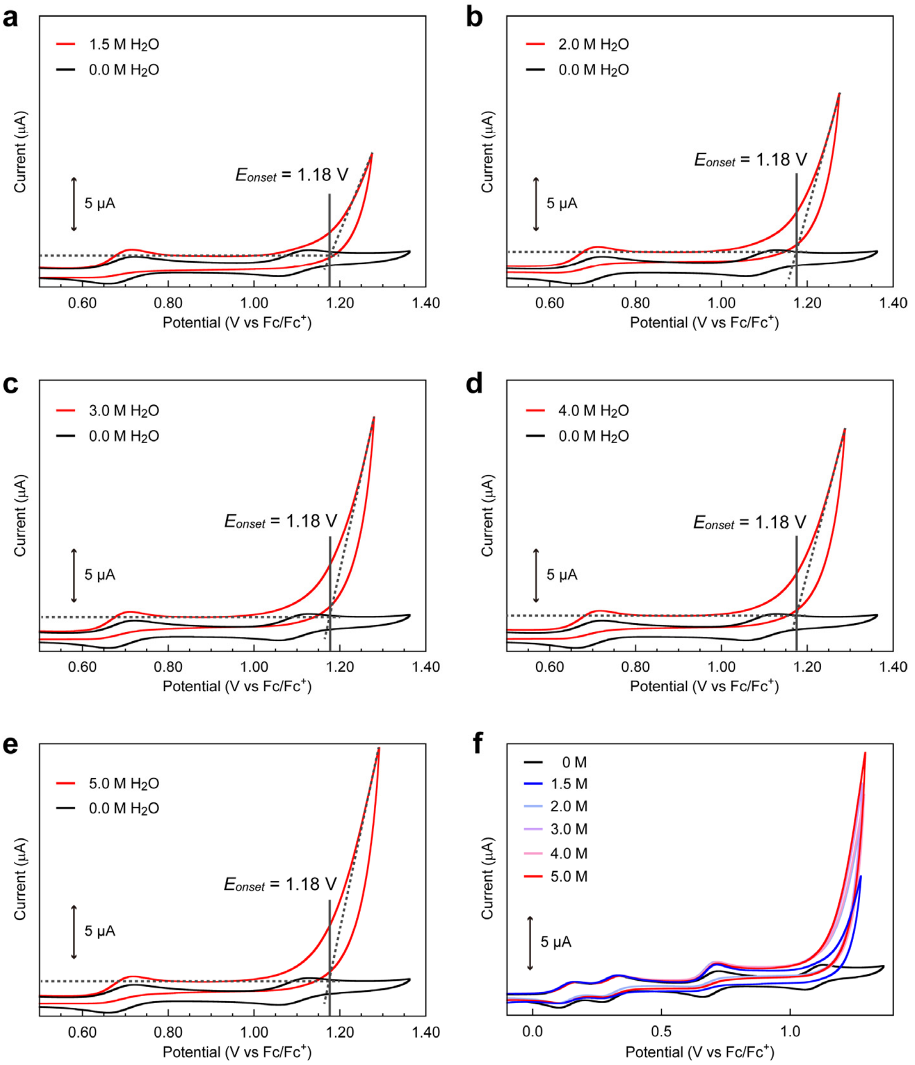


**Figure S16.** Cyclic voltammograms of **[Fe$_5$-H]$^{3+}$** (0.2 mM) in the presence of (a) 1.5, (b) 2.0, (c) 3.0, (d) 4.0, (e) 5.0 M of $H_2O$ (pH = 5, red lines) and in the absence of $H_2O$ (black lines). (f) Overlaid cyclic voltammograms of **[Fe$_5$-H]$^{3+}$** at various concentrations of $H_2O$. Cyclic voltammograms are measured in acetonitrile solutions with TBAP (0.1 M) on a GC electrode at a scan rate of 10 mV s$^{-1}$.

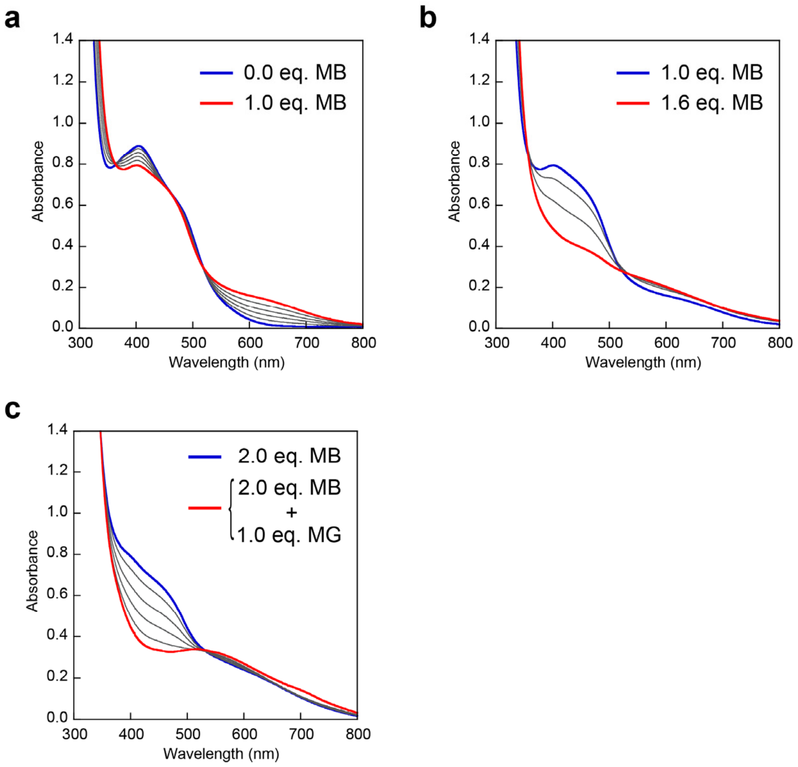


**Figure S17.** UV-Vis spectral changes of $\mathbf{[Fe_5\text{-}H]^{3+}}$ (0.05 mM) in MeCN upon addition of tris(4-bromophenyl)ammoniumyl hexachloroantimonate (Magic blue (**MB**), $E_{ox}$ = 0.67 V *vs*. Fc/Fc$^{+}$[S2]) and tris(2,4-dibromophenyl) ammoniumyl hexachloroantimonate (Magic green (MG), $E_{ox}$ = 1.14 V vs. Fc/Fc$^{+}$[S2]) as chemical oxidants at 293 K. (a) 0−1.0 eq of MB, (b) 1.0−1.6 eq. of MB and (c) 0−1.0 eq of MG after the addition of 2.0 eq. of MB. Reproduced with permission from Ref. S1. © 2016 Springer Nature.

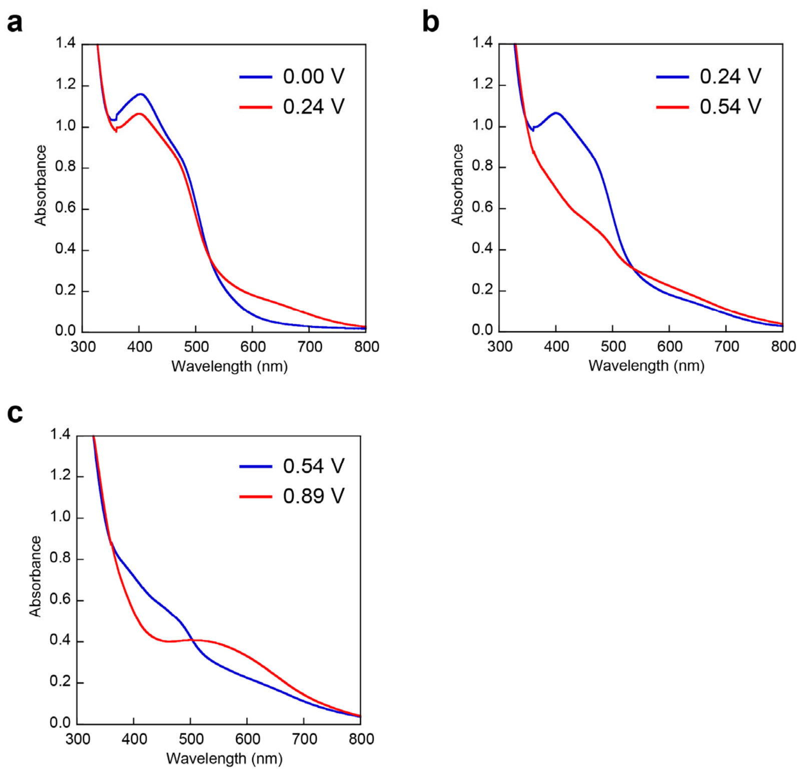


**Figure S18.** UV-Vis absorption spectra of **$[Fe_5\text{-}H]^{3+}$** (0.05 mM) at various applied potentials ((a) 0.00-0.24, (b) 0.24-0.54 and (c) 0.54-0.89 V(*vs*. $Fc/Fc^+$)) in 0.1 M TBAP/MeCN under Ar. Solutions were purged with Ar for 15 min prior to measurements. Weak Ar flow was maintained throughout the measurement.. Reproduced with permission from Ref. S1. © 2016 Springer Nature.

## Reaction Mechanism

1. Reaction mechanism of **$[Fe_5\text{-}H]^{3+}$** and **$[Fe_5\text{-}Me]^{3+}$**

So far, two different reaction pathways have been reported for water oxidation reaction catalysed by **$[Fe_5\text{-}H]^{3+}$** : the original mechanism in our first report (*Nature*, **2016**, *530*, 465., Path I, shown as Figure 5 in the main text) and the recently reported new mechanism based on computational study (*ACS Catal*. **2018**, *8*, 12, 11671. Path II). The difference between these two reaction mechanisms is the potentials required to drive the reaction. In Path I, four electron oxidized species (the **$S_4$** state) reacts with water, and the latter processes proceed without further electron transfer reactions. In contrast, in Path II, the **$S_4$** state reacts with water and the formed species is further oxidized at higher potential region. Both pathways are expected to proceed when substantially high potential is applied to the system. However, a profile of the CV of **$[Fe_5\text{-}H]^{3+}$** in the presence of $H_2O$ indicates that Path I is a dominant reaction pathway. Emergence of a catalytic current coupled with the formation of the **$S_4$** state suggests that further oxidation of **$S_4$** state is not necessarily required to drive the reaction. Therefore, at this stage, we believe that at lower potential region as we investigated in the present study, the reaction via Path I mainly proceeds and the reaction via Path II can be regarded as a minor pathway. From these reasons, we proposed the reaction pathway shown in Figure 5 to explain the reaction mechanism of the **$[Fe_5\text{-}H]^{3+}$** and **$[Fe_5\text{-}Me]^{3+}$**.

2. Reaction mechanism of **[Fe$_5$-Br]$^{3+}$**

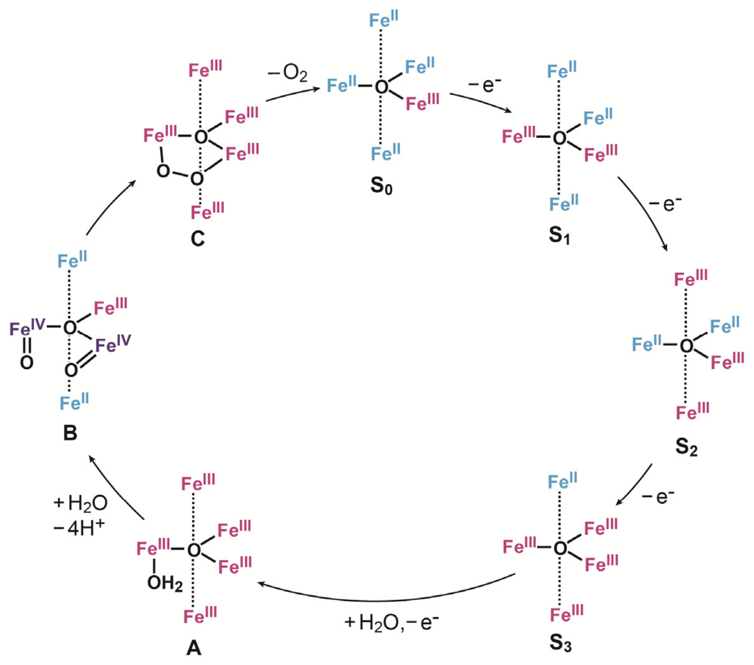


**Figure S19.** Plausible reaction mechanism for water oxidation catalysed by **[Fe$_5$-Br]$^{3+}$**. Fe$^{II}$, blue; Fe$^{III}$, red; and Fe$^{IV}$, purple.

## References


[S1] Okamura, M.; Kondo, M.; Kuga, R.; Kurashige, Y.; Yanai, T.; Hayami, S.; Praneeth, V. K. K.; Yoshida, M.; Yoneda, K.; Kawata, S.; Masaoka. S. A Pentanuclear Iron Catalyst Designed for Water Oxidation. *Nature* **2016**, *530*, 465–468.

[S2] Connelly, N. G. & Geiger, W. E. Chemical redox agents for organometallic chemistry. *Chem. Rev.* **96**, 877−910 (1996).

[S3] Gouré, E.; Gerey, B.; Clémancey, M.; Pécaut, J.; Molton, F.; Latour, J. M.; Blondin, G.; Collomb, M. N. Intramolecular Electron Transfers Thwart Bistability in a Pentanuclear Iron Complex. *Inorg. Chem.* **2016**, *55*, 9178–9186.


## Author Contributions

The manuscript was written through contributions of all authors. / All authors have given approval to the final version of the manuscript. / †These authors contributed equally.
